# Supplementary material for: Orientation control strategies and adaptation to a visuomotor perturbation in rotational hand movements
Source: PLoS Comput Biol. 2022 Dec 5;18(12):e1010248. doi: 10.1371/journal.pcbi.1010248 (PMC9754612; doi:10.1371/journal.pcbi.1010248)
Supplement: S1 Appendix — Tables A—C present the results of the sensitivity analysis that we performed on the number of averaged trials in the aiming axes analysis of experiment 2 (Table A—comparison within groups, Table B—comparisons between groups, and Table C—tests for the mean axis). Tables D—F present the results of the sensitivity analysis that we performed on the number of averaged trials in the aiming angles analysis of experiment 2 (Table D—ANOVA results, Table E—comparisons within groups, and Table F—comparisons between groups). *p < 0.05, **p < 0.001. (PDF) [file pcbi.1010248.s001.pdf]

**Table A. The results of the sensitivity analysis of the aiming axes - comparisons within groups.**

| | | Late BL1 vs. Early TRN | | | Early TRN vs. Late TRN | | | Late BL1 vs. Late TRN | | |
|---|---|---|---|---|---|---|---|---|---|---|
| **Group** | **n** | $\chi_2^2$ | **p** | **mean diff.** | $\chi_2^2$ | **p** | **mean diff.** | $\chi_2^2$ | **p** | **mean diff.** |
| Extrinsic | 1 | 17.9 | ** | 15.1° | 24.69 | ** | 22.29° | 42.28 | ** | 23.9° |
| | 2 | 20.93 | ** | 17.2° | 21.69 | ** | 20.7 ° | 48.11 | ** | 23.4° |
| | 3 | 37.06 | ** | 22.5° | 13.71 | * | 16.5 ° | 52.44 | ** | 22.7° |
| | 4 | 59.27 | ** | 27.5° | 9.43 | * | 13.6° | 41.79 | ** | 20.2° |
| | 5 | 84.4 | ** | 30° | 7.52 | * | 11.8° | 39.17 | ** | 19.7° |
| | 6 | 103.74 | ** | 32.3° | 4.62 | 0.1 | 8.8° | 46.37 | ** | 20.2° |
| | 7 | 121.02 | ** | 34° | 3.7 | 0.16 | 7.7° | 46.2 | ** | 19.7° |
| | 8 | 133.58 | ** | 36.2° | 2.26 | 0.32 | 6.1° | 46.47 | ** | 19.6° |
| | 9 | 139.38 | ** | 37.6° | 1.29 | 0.52 | 4.6° | 49.96 | ** | 19.7° |
| | 10 | 145.54 | ** | 38.5° | 0.84 | 0.66 | 3.7° | 55.3 | ** | 20° |
| Intrinsic | 1 | 1.3 | 0.52 | 9.4° | 24.13 | ** | 34.7° | 23.23 | ** | 19.2° |
| | 2 | 1.14 | 0.56 | 8.5° | 18.98 | ** | 25.5° | 26.56 | ** | 18.9° |
| | 3 | 5.54 | 0.06 | 15.1° | 13.9 | ** | 23.2° | 25.59 | ** | 20.4° |
| | 4 | 12.74 | ** | 18.5° | 16.95 | ** | 21.5° | 30.82 | ** | 19.8° |
| | 5 | 17.28 | ** | 20.3° | 15.63 | ** | 21.5° | 39.17 | ** | 19.8° |
| | 6 | 22.63 | ** | 22.8° | 15.71 | ** | 21.3° | 45.51 | ** | 19.6° |
| | 7 | 30.44 | ** | 25.2° | 14.45 | ** | 19.5° | 50.51 | ** | 19.9° |
| | 8 | 36.8 | ** | 26.6° | 14.8 | ** | 18.9° | 59.36 | ** | 20.2° |
| | 9 | 38.55 | ** | 27.3° | 13.81 | * | 18.2° | 67.33 | ** | 20.7° |
| | 10 | 45.07 | ** | 28.8° | 13.79 | * | 17.7° | 71 | ** | 20.6° |
| Control | 1 | 1.2 | 0.55 | 7.1° | 33.97 | ** | 41.5° | 37.7 | ** | 24.1° |
| | 2 | 9.58 | ** | 13° | 33.77 | ** | 27.4° | 35.55 | ** | 20.6° |
| | 3 | 3.86 | ** | 19.4° | 20.77 | ** | 19.8° | 31.63 | ** | 21.5° |
| | 4 | 59.3 | ** | 23.8° | 14.92 | ** | 16.4° | 37.32 | ** | 21.2° |
| | 5 | 56.33 | ** | 24.9° | 14.67 | ** | 16.7° | 33.63 | ** | 20.4° |
| | 6 | 66.83 | ** | 25.9° | 14.69 | ** | 16.3° | 38.1 | ** | 20.4° |
| | 7 | 66.51 | ** | 25.9° | 13.9 | ** | 16.1° | 36.72 | ** | 20.7° |
| | 8 | 58.6 | ** | 27° | 12.34 | * | 15.7° | 36.21 | ** | 20.3° |
| | 9 | 61.7 | ** | 27.2° | 11.54 | * | 15.2° | 36.9 | ** | 20.5° |
| | 10 | 64.97 | ** | 27.2° | 12.55 | * | 15.2° | 36.02 | ** | 20.2° |

| | | Late BL2 vs. Early TFR | | | Early TFR vs. Late TFR | | | Late BL2 vs. Late TFR | | |
|---|---|---|---|---|---|---|---|---|---|---|
| **Group** | **n** | $\chi^2_2$ | **p** | **mean diff.** | $\chi^2_2$ | **p** | **mean diff.** | $\chi^2_2$ | **p** | **mean diff.** |
| Extrinsic | 1 | 38.12 | ** | 26.3° | 23.25 | ** | 22.3° | 1.11 | 0.57 | 4.8° |
| | 2 | 40.78 | ** | 21.7° | 21.2 | ** | 17.4° | 1.73 | 0.42 | 5.1° |
| | 3 | 52.46 | ** | 19.3° | 32.2 | ** | 18.2° | 0.47 | 0.79 | 2.4° |
| | 4 | 62.36 | ** | 17.6° | 27.23 | ** | 16.2° | 0.23 | 0.89 | 1.6° |
| | 5 | 61.34 | ** | 17.7° | 22.6 | ** | 15.1° | 1.01 | 0.6 | 3.4° |
| | 6 | 49.64 | ** | 16.9° | 18.83 | ** | 14.7° | 0.47 | 0.79 | 2.3° |
| | 7 | 45.69 | ** | 15.9° | 17 | ** | 13.7° | 0.55 | 0.76 | 2.3° |
| | 8 | 43.69 | ** | 15.2° | 16.87 | ** | 13.3° | 0.53 | 0.77 | 2.3° |
| | 9 | 36.57 | ** | 13.8° | 14.64 | ** | 12° | 0.82 | 0.66 | 2.7° |
| | 10 | 33.03 | ** | 13.8° | 15.37 | ** | 12.1° | 1.06 | 0.59 | 2.9° |
| Intrinsic | 1 | 0.83 | 0.66 | 4.3° | 2.13 | 0.34 | 12.4° | 4.67 | 0.1 | 8.5° |
| | 2 | 5.03 | 0.08 | 6.2° | 1.32 | 0.52 | 5.2° | 7.13 | * | 9.6° |
| | 3 | 7.06 | * | 6.6° | 1.51 | 0.47 | 3.7° | 6.99 | * | 8.3° |
| | 4 | 7.68 | * | 6.6° | 1.16 | 0.56 | 2° | 5.22 | 0.07 | 6.8° |
| | 5 | 7.45 | * | 6.1° | 0.77 | 0.68 | 2.2° | 5.58 | 0.06 | 7° |
| | 6 | 7.45 | * | 5.5° | 0.52 | 0.77 | 2° | 4.95 | 0.08 | 6.6° |
| | 7 | 7.33 | * | 5.5° | 0.5 | 0.78 | 2.6° | 6.22 | * | 7.5° |
| | 8 | 6.37 | * | 5° | 0.25 | 0.88 | 1.9° | 4.92 | 0.08 | 6.5° |
| | 9 | 4.91 | 0.09 | 4.5° | 0.19 | 0.9 | 2.1° | 4.01 | 0.13 | 5.7° |
| | 10 | 5.85 | 0.05 | 4.9° | 0.24 | 0.88 | 1.8° | 3.57 | 0.17 | 5.3° |
| Control | 1 | 5.56 | 0.06 | 9.8° | 0.52 | 0.77 | 3.6° | 4.72 | 0.1 | 7.5° |
| | 2 | 6.64 | * | 11.5° | 0.66 | 0.72 | 3.2° | 7.13 | * | 10.6° |
| | 3 | 7.91 | * | 13.8° | 1.29 | 0.52 | 3.9° | 6.1 | * | 11.6° |
| | 4 | 9.32 | * | 11.5° | 0.99 | 0.61 | 3.3° | 7.11 | * | 9.7° |
| | 5 | 9.43 | * | 12.7° | 1.09 | 0.58 | 3.3° | 7.81 | * | 11.6° |
| | 6 | 11.06 | * | 11.4° | 1.64 | 0.44 | 4° | 7.77 | * | 9.7° |
| | 7 | 12.77 | * | 11° | 1.24 | 0.54 | 3.4° | 8.49 | * | 9.6° |
| | 8 | 14.61 | ** | 10.5° | 2.05 | 0.36 | 4° | 9.28 | * | 8.4° |
| | 9 | 13.44 | * | 10.9° | 1.61 | 0.44 | 3.4° | 9.17 | * | 9° |
| | 10 | 15.3 | ** | 10.3° | 1.14 | 0.56 | 2.8° | 10.46 | * | 8.9° |

| | Extrinsic Group: Late TRN vs. Early TFR | | | | | | | | | |
|---|---|---|---|---|---|---|---|---|---|---|
| **n** | 1 | 2 | 3 | 4 | 5 | 6 | 7 | 8 | 9 | 10 |
| $\chi^2_2$ | 22.65 | 35.44 | 50.59 | 64.5 | 66.5 | 66.6 | 68.81 | 68.84 | 74.15 | 71.1 |
| **p** | ** | ** | ** | ** | ** | ** | ** | ** | ** | ** |
| **mean diff.** | 26.6° | 26.5° | 26.5° | 28.2° | 29.1° | 28.8° | 29.5° | 29.7° | 30.6° | 30.2° |

**Table B. The results of the sensitivity analysis of the aiming axes - comparisons between groups.**

| | | Extrinsic vs. Intrinsic | | | Extrinsic vs. Control | | | Intrinsic vs. Control | | |
|---|---|---|---|---|---|---|---|---|---|---|
| **Stage** | **n** | $\chi_2^2$ | **p** | **mean diff.** | $\chi_2^2$ | **p** | **mean diff.** | $\chi_2^2$ | **p** | **mean diff.** |
| Early TRN | 1 | 10.36 | * | 22.5° | 13.19 | * | 24.5° | 0.52 | 0.77 | 5.1° |
| | 2 | 6.27 | 0.05 | 17.6° | 5.34 | 0.07 | 10.6° | 1.29 | 0.52 | 6.9° |
| | 3 | 5.89 | * | 14.6° | 5.62 | 0.06 | 8.5° | 1.25 | 0.53 | 6.3° |
| | 4 | 9.55 | * | 15.4° | 5.89 | 0.05 | 8° | 2.11 | 0.35 | 7.3° |
| | 5 | 10.45 | * | 15.1° | 8.04 | * | 9° | 1.41 | 0.49 | 6.1° |
| | 6 | 12.49 | * | 17.2° | 11.72 | * | 10.4° | 1.58 | 0.45 | 6.9° |
| | 7 | 13.05 | * | 16.7° | 13.79 | * | 11.3° | 1.69 | 0.43 | 6.2° |
| | 8 | 15.48 | * | 17.6° | 14.12 | ** | 12.1° | 2.9 | 0.39 | 6.7° |
| | 9 | 15.88 | * | 18.2° | 15.84 | ** | 12.8° | 2.27 | 0.32 | 7.5° |
| | 10 | 15.9 | * | 18.2° | 17.71 | ** | 13.4° | 2.52 | 0.28 | 7.8° |
| Late TRN | 1 | 1.1 | 0.58 | 4.3° | 1.5 | 0.47 | 5.8° | 0.14 | 0.93 | 1.6° |
| | 2 | 0.91 | 0.63 | 3.7° | 0.76 | 0.68 | 3.5° | 0.22 | 0.9 | 1.8° |
| | 3 | 1.67 | 0.43 | 5.6° | 1.29 | 0.52 | 5.1° | 0.17 | 0.92 | 2° |
| | 4 | 2.04 | 0.36 | 5.9° | 1.32 | 0.52 | 5° | 0.29 | 0.87 | 2.3° |
| | 5 | 1.04 | 0.38 | 5.7° | 0.83 | 0.66 | 4° | 0.38 | 0.82 | 2.6° |
| | 6 | 1.33 | 0.51 | 4.5° | 0.45 | 0.8 | 2.8° | 0.37 | 0.83 | 2.5° |
| | 7 | 1.27 | 0.53 | 4.2° | 0.44 | 0.8 | 2.8° | 0.41 | 0.82 | 2.6° |
| | 8 | 1.46 | 0.48 | 4.5° | 0.37 | 0.83 | 2.5° | 0.42 | 0.81 | 2.6° |
| | 9 | 1.52 | 0.47 | 4.4° | 0.31 | 0.85 | 2.3° | 0.47 | 0.79 | 2.7° |
| | 10 | 1.35 | 0.51 | 4° | 0.12 | 0.94 | 1.4° | 0.6 | 0.84 | 3° |
| Early TFR | 1 | 5.54 | 0.06 | 17.1° | 39.16 | ** | 30.3° | 5.18 | 0.07 | 13.9° |
| | 2 | 9.41 | * | 15.2° | 38.4 | ** | 24.3° | 7.23 | * | 9.9° |
| | 3 | 16 | ** | 14.9° | 63.94 | ** | 24.8° | 12.18 | ** | 11.2° |
| | 4 | 20.72 | ** | 14.4° | 71.07 | ** | 23.5° | 14 | ** | 10.9° |
| | 5 | 21.79 | ** | 13.7° | 72.54 | ** | 22.7° | 13.81 | ** | 10.2° |
| | 6 | 20.84 | ** | 13.2° | 61.03 | ** | 22.2° | 14.71 | ** | 9.8° |
| | 7 | 18.16 | ** | 12.4° | 61.01 | ** | 21.4° | 15.51 | ** | 9.8° |
| | 8 | 19.49 | ** | 12.3° | 62.01 | ** | 21.4° | 15.52 | ** | 9.7° |
| | 9 | 16.93 | ** | 10.8° | 53.86 | ** | 19.9° | 13.38 | ** | 9.6° |
| | 10 | 16.61 | ** | 10.8° | 52.51 | ** | 19.7° | 14.58 | ** | 9.6° |

**Table C. The results of the sensitivity analysis of the aiming axes - tests for mean axis.**

| | | **Ideal BL1 vs. Early TRN** | | | | **Rotated Ideal BL1 vs. Late TRN** | | | |
|---|---|---|---|---|---|---|---|---|---|
| **Group** | **n** | $\mu$ | $\kappa$ | **p** | **mean diff.** | $\mu$ | $\kappa$ | **p** | **mean diff.** |
| Extrinsic | 1 | $0.09\hat{\mathbf{i}} - 0.04\hat{\mathbf{j}} + 0.91\hat{\mathbf{k}}$ | 31 | * | 21.5° | $0.13\hat{\mathbf{i}} - 0.02\hat{\mathbf{j}} + 0.99\hat{\mathbf{k}}$ | 31.22 | * | 17.72° |
| | 2 | $0.07\hat{\mathbf{i}} - 0.39\hat{\mathbf{j}} + 0.92\hat{\mathbf{k}}$ | 30.17 | * | 22.47° | $0.11\hat{\mathbf{i}} - 0.04\hat{\mathbf{j}} + 0.99\hat{\mathbf{k}}$ | 40 | * | 18.27° |
| | 3 | $0.08\hat{\mathbf{i}} - 0.32\hat{\mathbf{j}} + 0.94\hat{\mathbf{k}}$ | 28.74 | * | 26.61° | $0.11\hat{\mathbf{i}} - 0.04\hat{\mathbf{j}} + 0.99\hat{\mathbf{k}}$ | 41.65 | * | 18.41° |
| | 4 | $0.07\hat{\mathbf{i}} - 0.26\hat{\mathbf{j}} + 0.96\hat{\mathbf{k}}$ | 29.98 | * | 30.24° | $0.11\hat{\mathbf{i}} - 0.03\hat{\mathbf{j}} + 0.99\hat{\mathbf{k}}$ | 40.15 | * | 17.78° |
| | 5 | $0.06\hat{\mathbf{i}} - 0.22\hat{\mathbf{j}} + 0.97\hat{\mathbf{k}}$ | 35.04 | * | 32.62° | $0.11\hat{\mathbf{i}} - 0.02\hat{\mathbf{j}} + 0.99\hat{\mathbf{k}}$ | 37.52 | * | 17.3° |
| | 6 | $0.07\hat{\mathbf{i}} - 0.18\hat{\mathbf{j}} + 0.98\hat{\mathbf{k}}$ | 37.78 | * | 34.76° | $0.11\hat{\mathbf{i}} - 0.03\hat{\mathbf{j}} + 0.99\hat{\mathbf{k}}$ | 42.99 | * | 18.11° |
| | 7 | $0.08\hat{\mathbf{i}} - 0.16\hat{\mathbf{j}} + 0.98\hat{\mathbf{k}}$ | 39.35 | * | 35.82° | $0.11\hat{\mathbf{i}} - 0.03\hat{\mathbf{j}} + 0.99\hat{\mathbf{k}}$ | 44.48 | * | 18.04° |
| | 8 | $0.08\hat{\mathbf{i}} - 0.13\hat{\mathbf{j}} + 0.99\hat{\mathbf{k}}$ | 37.99 | * | 37.65° | $0.11\hat{\mathbf{i}} - 0.03\hat{\mathbf{j}} + 0.99\hat{\mathbf{k}}$ | 44.51 | * | 18.07° |
| | 9 | $0.08\hat{\mathbf{i}} - 0.11\hat{\mathbf{j}} + 0.99\hat{\mathbf{k}}$ | 36.38 | * | 39.11° | $0.11\hat{\mathbf{i}} - 0.03\hat{\mathbf{j}} + 0.99\hat{\mathbf{k}}$ | 47 | * | 18.09° |
| | 10 | $0.08\hat{\mathbf{i}} - 0.09\hat{\mathbf{j}} + 0.99\hat{\mathbf{k}}$ | 36.07 | * | 39.85° | $0.11\hat{\mathbf{i}} - 0.04\hat{\mathbf{j}} + 0.99\hat{\mathbf{k}}$ | 50.15 | * | 18.57° |
| Intrinsic | 1 | $-0.11\hat{\mathbf{i}} + 0.72\hat{\mathbf{j}} + 0.68\hat{\mathbf{k}}$ | 6.64 | 0.66 | 6.65° | $0.1\hat{\mathbf{i}} + 0.99\hat{\mathbf{j}} + 0.1\hat{\mathbf{k}}$ | 53.8 | * | 21.05° |
| | 2 | $-0.06\hat{\mathbf{i}} + 0.78\hat{\mathbf{j}} + 0.63\hat{\mathbf{k}}$ | 6.89 | 0.65 | 6.94° | $0.06\hat{\mathbf{i}} + 0.99\hat{\mathbf{j}} + 0.08\hat{\mathbf{k}}$ | 52.9 | * | 20.13° |
| | 3 | $-0.04\hat{\mathbf{i}} + 0.85\hat{\mathbf{j}} + 0.52\hat{\mathbf{k}}$ | 10.21 | 0.13 | 13.55° | $0.05\hat{\mathbf{i}} + 0.99\hat{\mathbf{j}} + 0.11\hat{\mathbf{k}}$ | 31.5 | * | 21.89° |
| | 4 | $-0.06\hat{\mathbf{i}} + 0.88\hat{\mathbf{j}} + 0.47\hat{\mathbf{k}}$ | 15.35 | * | 16.89° | $0.05\hat{\mathbf{i}} + 0.99\hat{\mathbf{j}} + 0.1\hat{\mathbf{k}}$ | 38.25 | * | 21.13° |
| | 5 | $-0.06\hat{\mathbf{i}} + 0.9\hat{\mathbf{j}} + 0.43\hat{\mathbf{k}}$ | 16.9 | * | 19.44° | $0.05\hat{\mathbf{i}} + 0.99\hat{\mathbf{j}} + 0.09\hat{\mathbf{k}}$ | 43.08 | * | 20.53° |
| | 6 | $-0.11\hat{\mathbf{i}} + 0.91\hat{\mathbf{j}} + 0.4\hat{\mathbf{k}}$ | 16.26 | * | 22° | $0.\hat{0}5\mathbf{i} + 0.99\hat{\mathbf{j}} + 0.09\hat{\mathbf{k}}$ | 47.02 | * | 20.41° |
| | 7 | $-0.13\hat{\mathbf{i}} + 0.92\hat{\mathbf{j}} + 0.36\hat{\mathbf{k}}$ | 17.91 | * | 24.85° | $0.06\hat{\mathbf{i}} + 0.99\hat{\mathbf{j}} + 0.09\hat{\mathbf{k}}$ | 50.18 | * | 20.2° |
| | 8 | $-0.14\hat{\mathbf{i}} + 0.93\hat{\mathbf{j}} + 0.34\hat{\mathbf{k}}$ | 19.21 | * | 26.28° | $0.06\hat{\mathbf{i}} + 0.99\hat{\mathbf{j}} + 0.09\hat{\mathbf{k}}$ | 55.78 | * | 20.48° |
| | 9 | $-0.15\hat{\mathbf{i}} + 0.93\hat{\mathbf{j}} + 0.32\hat{\mathbf{k}}$ | 18.89 | * | 27.33° | $0.06\hat{\mathbf{i}} + 0.99\hat{\mathbf{j}} + 0.09\hat{\mathbf{k}}$ | 59.82 | * | 20.65° |
| | 10 | $-0.16\hat{\mathbf{i}} + 0.94\hat{\mathbf{j}} + 0.3\hat{\mathbf{k}}$ | 19.82 | * | 28.75° | $0.07\hat{\mathbf{i}} + 0.99\hat{\mathbf{j}} + 0.09\hat{\mathbf{k}}$ | 62.75 | * | 20.64° |
| Control | 1 | $-0.05\hat{\mathbf{i}} + 0.68\hat{\mathbf{j}} + 0.73\hat{\mathbf{k}}$ | 9.58 | 0.86 | 3.73° | $0.08\hat{\mathbf{i}} + 0.99\hat{\mathbf{j}} + 0.11\hat{\mathbf{k}}$ | 29.61 | * | 21.81° |
| | 2 | $-0.01\hat{\mathbf{i}} + 0.84\hat{\mathbf{j}} + 0.54\hat{\mathbf{k}}$ | 24.55 | * | 12.54° | $0.09\hat{\mathbf{i}} + 0.99\hat{\mathbf{j}} + 0.1\hat{\mathbf{k}}$ | 41.22 | * | 21.25° |
| | 3 | $0.03\hat{\mathbf{i}} + 0.89\hat{\mathbf{j}} + 0.45\hat{\mathbf{k}}$ | 47.83 | * | 18.36° | $0.09\hat{\mathbf{i}} + 0.99\hat{\mathbf{j}} + 0.12\hat{\mathbf{k}}$ | 28.62 | * | 22.64° |
| | 4 | $0.93\hat{\mathbf{j}} + 0.37\hat{\mathbf{k}}$ | 43.32 | * | 23.08° | $0.09\hat{\mathbf{i}} + 0.99\hat{\mathbf{j}} + 0.11\hat{\mathbf{k}}$ | 32.97 | * | 21.8° |
| | 5 | $-0.01\hat{\mathbf{i}} + 0.94\hat{\mathbf{j}} + 0.35\hat{\mathbf{k}}$ | 38.08 | * | 24.54° | $0.09\hat{\mathbf{i}} + 0.99\hat{\mathbf{j}} + 0.08\hat{\mathbf{k}}$ | 33.2 | * | 20.57° |
| | 6 | $-0.03\hat{\mathbf{i}} + 0.94\hat{\mathbf{j}} + 0.33\hat{\mathbf{k}}$ | 39.4 | * | 25.79° | $0.1\hat{\mathbf{i}} + 0.99\hat{\mathbf{j}} + 0.08\hat{\mathbf{k}}$ | 35.43 | * | 20.48° |
| | 7 | $-0.03\hat{\mathbf{i}} + 0.94\hat{\mathbf{j}} + 0.32\hat{\mathbf{k}}$ | 39.38 | * | 26.08° | $0.1\hat{\mathbf{i}} + 0.99\hat{\mathbf{j}} + 0.08\hat{\mathbf{k}}$ | 33.5 | * | 20.53° |
| | 8 | $-0.03\hat{\mathbf{i}} + 0.95\hat{\mathbf{j}} + 0.31\hat{\mathbf{k}}$ | 32.47 | * | 27.09° | $0.1\hat{\mathbf{i}} + 0.99\hat{\mathbf{j}} + 0.08\hat{\mathbf{k}}$ | 35.48 | * | 20.2° |
| | 9 | $-0.02\hat{\mathbf{i}} + 0.95\hat{\mathbf{j}} + 0.3\hat{\mathbf{k}}$ | 32 | * | 27.42° | $0.11\hat{\mathbf{i}} + 0.99\hat{\mathbf{j}} + 0.07\hat{\mathbf{k}}$ | 34.83 | * | 20.14° |
| | 10 | $-0.02\hat{\mathbf{i}} + 0.95\hat{\mathbf{j}} + 0.3\hat{\mathbf{k}}$ | 34.33 | * | 27.49° | $0.11\hat{\mathbf{i}} + 0.99\hat{\mathbf{j}} + 0.07\hat{\mathbf{k}}$ | 34.38 | * | 19.81° |

| | | Ideal BL2 vs. Early TFR | | | | Ideal BL2 vs. Late TFR | | | |
|---|---|---|---|---|---|---|---|---|---|
| **Group** | **n** | $\mu$ | $\kappa$ | **p** | **mean diff.** | $\mu$ | $\kappa$ | **p** | **mean diff.** |
| Extrinsic | 1 | $-0.04\hat{\mathbf{i}} + 0.93\hat{\mathbf{j}} + 0.36\hat{\mathbf{k}}$ | 35.22 | * | 22.91° | $-0.03\hat{\mathbf{i}} + 0.72\hat{\mathbf{j}} + 0.69\hat{\mathbf{k}}$ | 29.22 | 0.73 | 2.44° |
| | 2 | $-0.03\hat{\mathbf{i}} + 0.91\hat{\mathbf{j}} + 0.42\hat{\mathbf{k}}$ | 49.25 | * | 18.8° | $-0.01\hat{\mathbf{i}} + 0.7\hat{\mathbf{j}} + 0.71\hat{\mathbf{k}}$ | 35.4 | 0.77 | 1.98° |
| | 3 | $-0.02\hat{\mathbf{i}} + 0.9\hat{\mathbf{j}} + 0.43\hat{\mathbf{k}}$ | 98.97 | * | 17.96° | $0.71\hat{\mathbf{j}} + 0.7\hat{\mathbf{k}}$ | 40.61 | 0.95 | 0.76° |
| | 4 | -0.02$\hat{\mathbf{i}} + 0.89\hat{\mathbf{j}} + 0.46\hat{\mathbf{k}}$ | 138.62 | * | 16.68° | $-0.02\hat{\mathbf{i}} + 0.71\hat{\mathbf{j}} + 0.7\hat{\mathbf{k}}$ | 41.19 | 0.96 | 0.58° |
| | 5 | $0.88\hat{\mathbf{j}} + 0.47\hat{\mathbf{k}}$ | 133.61 | * | 15.83° | $0.71\hat{\mathbf{j}} + 0.7\hat{\mathbf{k}}$ | 39.45 | 0.81 | 1.53° |
| | 6 | $0.87\hat{\mathbf{j}} + 0.49\hat{\mathbf{k}}$ | 94.96 | * | 15.07° | $0.71\hat{\mathbf{j}} + 0.7\hat{\mathbf{k}}$ | 39.29 | 0.97 | 0.5° |
| | 7 | $0.01\hat{\mathbf{i}} + 0.87\hat{\mathbf{j}} + 0.5\hat{\mathbf{k}}$ | 88.32 | * | 14.27° | $0.01\hat{\mathbf{i}} + 0.71\hat{\mathbf{j}} + 0.7\hat{\mathbf{k}}$ | 41.23 | 0.95 | 0.69° |
| | 8 | $0.02\hat{\mathbf{i}} + 0.87\hat{\mathbf{j}} + 0.5\hat{\mathbf{k}}$ | 86.98 | * | 14.03° | $0.01\hat{\mathbf{i}} + 0.72\hat{\mathbf{j}} + 0.7\hat{\mathbf{k}}$ | 43.07 | 0.9 | 0.92° |
| | 9 | $0.02\hat{\mathbf{i}} + 0.86\hat{\mathbf{j}} + 0.51\hat{\mathbf{k}}$ | 81.38 | * | 12.93° | $0.01\hat{\mathbf{i}} + 0.72\hat{\mathbf{j}} + 0.7\hat{\mathbf{k}}$ | 53.08 | 0.75 | 1.65° |
| | 10 | $0.02\hat{\mathbf{i}} + 0.86\hat{\mathbf{j}} + 0.51\hat{\mathbf{k}}$ | 73.62 | * | 12.94° | $0.02\hat{\mathbf{i}} + 0.72\hat{\mathbf{j}} + 0.7\hat{\mathbf{k}}$ | 53.08 | 0.75 | 1.65° |
| Intrinsic | 1 | $-0.04\hat{\mathbf{i}} + 0.79\hat{\mathbf{j}} + 0.6\hat{\mathbf{k}}$ | 11.56 | 0.39 | 8.38° | $0.09\hat{\mathbf{i}} + 0.73\hat{\mathbf{j}} + 0.68\hat{\mathbf{k}}$ | 39.32 | 0.22 | 5.55° |
| | 2 | $0.05\hat{\mathbf{i}} + 0.78\hat{\mathbf{j}} + 0.62\hat{\mathbf{k}}$ | 27.31 | 0.21 | 4.1° | $0.1\hat{\mathbf{i}} + 0.73\hat{\mathbf{j}} + 0.68\hat{\mathbf{k}}$ | 41.41 | 0.17 | 6.08° |
| | 3 | $0.06\hat{\mathbf{i}} + 0.77\hat{\mathbf{j}} + 0.63\hat{\mathbf{k}}$ | 42.41 | 0.13 | 4.1° | $0.08\hat{\mathbf{i}} + 0.72\hat{\mathbf{j}} + 0.69\hat{\mathbf{k}}$ | 52.25 | 0.22 | 5.02° |
| | 4 | $0.07\hat{\mathbf{i}} + 0.76\hat{\mathbf{j}} + 0.64\hat{\mathbf{k}}$ | 57.55 | 0.1 | 4.05° | $0.06\hat{\mathbf{i}} + 0.72\hat{\mathbf{j}} + 0.69\hat{\mathbf{k}}$ | 60.49 | 0.36 | 3.64° |
| | 5 | $0.06\hat{\mathbf{i}} + 0.75\hat{\mathbf{j}} + 0.66\hat{\mathbf{k}}$ | 69.83 | 0.17 | 3.36° | $0.07\hat{\mathbf{i}} + 0.72\hat{\mathbf{j}} + 0.69\hat{\mathbf{k}}$ | 57.09 | 0.33 | 3.96° |
| | 6 | $0.06\hat{\mathbf{i}} + 0.74\hat{\mathbf{j}} + 0.66\hat{\mathbf{k}}$ | 91.28 | 0.14 | 2.94° | $0.06\hat{\mathbf{i}} + 0.72\hat{\mathbf{j}} + 0.69\hat{\mathbf{k}}$ | 55.79 | 0.32 | 3.89° |
| | 7 | $0.05\hat{\mathbf{i}} + 0.74\hat{\mathbf{j}} + 0.67\hat{\mathbf{k}}$ | 90.44 | 0.16 | 2.91° | $0.08\hat{\mathbf{i}} + 0.71\hat{\mathbf{j}} + 0.69\hat{\mathbf{k}}$ | 50.95 | 0.26 | 4.67° |
| | 8 | $0.06\hat{\mathbf{i}} + 0.74\hat{\mathbf{j}} + 0.67\hat{\mathbf{k}}$ | 95.16 | 0.18 | 2.78° | $0.07\hat{\mathbf{i}} + 0.72\hat{\mathbf{j}} + 0.7\hat{\mathbf{k}}$ | 54.74 | 0.32 | 3.99° |
| | 9 | $0.05\hat{\mathbf{i}} + 0.73\hat{\mathbf{j}} + 0.68\hat{\mathbf{k}}$ | 95.93 | 0.25 | 2.89° | $0.06\hat{\mathbf{i}} + 0.72\hat{\mathbf{j}} + 0.69\hat{\mathbf{k}}$ | 56.96 | 0.36 | 3.69° |
| | 10 | $0.06\hat{\mathbf{i}} + 0.73\hat{\mathbf{j}} + 0.68\hat{\mathbf{k}}$ | 100.43 | 0.17 | 3.2° | $0.06\hat{\mathbf{i}} + 0.71\hat{\mathbf{j}} + 0.7\hat{\mathbf{k}}$ | 59.4 | 0.38 | 3.34° |
| Control | 1 | $-0.8\hat{\mathbf{j}} + 0.6\hat{\mathbf{k}}$ | 22.84 | 0.19 | 7.9° | $-0.05\hat{\mathbf{i}} - 0.77\hat{\mathbf{j}} + 0.64\hat{\mathbf{k}}$ | 35.25 | 0.26 | 6.21° |
| | 2 | $-0.01\hat{\mathbf{i}} - 0.77\hat{\mathbf{j}}0.63\hat{\mathbf{k}}$ | 34.26 | 0.24 | 5.78° | $-0.06\hat{\mathbf{i}} - 0.76\hat{\mathbf{j}} + 0.65\hat{\mathbf{k}}$ | 57.36 | 0.17 | 5.62° |
| | 3 | $-0.03\hat{\mathbf{i}} - 0.79\hat{\mathbf{j}} + 0.61\hat{\mathbf{k}}$ | 47.29 | 0.09 | 7.17° | $-0.07\hat{\mathbf{i}} - 0.75\hat{\mathbf{j}} + 0.66\hat{\mathbf{k}}$ | 67.49 | 0.12 | 5.59° |
| | 4 | $-0.03\hat{\mathbf{i}} - 0.79\hat{\mathbf{j}} - 0.61\hat{\mathbf{k}}$ | 56.95 | 0.06 | 7.18° | $-0.07\hat{\mathbf{i}} - 0.76\hat{\mathbf{j}} + 0.65\hat{\mathbf{k}}$ | 66.43 | 0.09 | 5.98° |
| | 5 | $-0.03\hat{\mathbf{i}} - 0.79\hat{\mathbf{j}} + 0.62\hat{\mathbf{k}}$ | 68.3 | 0.05 | 7.06° | $-0.07\hat{\mathbf{i}} - 0.76\hat{\mathbf{j}} + 0.64\hat{\mathbf{k}}$ | 66.55 | 0.08 | 6.42° |
| | 6 | $-0.02\hat{\mathbf{i}} - 0.79\hat{\mathbf{j}} + 0.61\hat{\mathbf{k}}$ | 71.19 | * | 7.26° | $-0.07\hat{\mathbf{i}} - 0.76\hat{\mathbf{j}} + 0.65\hat{\mathbf{k}}$ | 67.18 | 0.06 | 6.1° |
| | 7 | $-0.02\hat{\mathbf{i}} - 0.79\hat{\mathbf{j}} + 0.61\hat{\mathbf{k}}$ | 84.25 | * | 7.24° | $-0.07\hat{\mathbf{i}} - 0.76\hat{\mathbf{j}} + 0.64\hat{\mathbf{k}}$ | 64.16 | 0.08 | 6.31° |
| | 8 | $-0.02\hat{\mathbf{i}} - 0.79\hat{\mathbf{j}} + 0.61\hat{\mathbf{k}}$ | 86.4 | * | 7.4° | $-0.07\hat{\mathbf{i}} - 0.76\hat{\mathbf{j}} + 0.65\hat{\mathbf{k}}$ | 87.41 | 0.05 | 5.83° |
| | 9 | $-0.02\hat{\mathbf{i}} - 0.79\hat{\mathbf{j}} + 0.62\hat{\mathbf{k}}$ | 92.27 | * | 6.98° | $-0.06\hat{\mathbf{i}} - 0.76\hat{\mathbf{j}} + 0.65\hat{\mathbf{k}}$ | 91.5 | 0.05 | 5.74° |
| | 10 | $-0.02\hat{\mathbf{i}} - 0.78\hat{\mathbf{j}} + 0.62\hat{\mathbf{k}}$ | 101.49 | * | 6.77° | $-0.06\hat{\mathbf{i}} - 0.76\hat{\mathbf{j}} + 0.65\hat{\mathbf{k}}$ | 87.13 | 0.05 | 5.9° |

**Table D. The results of the sensitivity analysis of the aiming angles - ANOVA table.**

| | Group | | | Stage | | | Group × Stage | | |
|---|---|---|---|---|---|---|---|---|---|
| **n** | $\mathbf{F_{2,27}}$ | **p** | $\eta_p^2$ | $\mathbf{F_{5,135}}$ | **p** | $\eta_p^2$ | $\mathbf{F_{10,135}}$ | **p** | $\eta_p^2$ |
| 1 | 7.81 | * | 0.37 | 34.81 | ** | 0.56 | 2.36 | * | 0.15 |
| 2 | 6.04 | * | 0.31 | 43.16 | ** | 0.62 | 2.11 | * | 0.13 |
| 3 | 5.45 | * | 0.29 | 51.2 | ** | 0.65 | 2.26 | * | 0.14 |
| 4 | 5.63 | * | 0.29 | 77.22 | ** | 0.74 | 2.59 | * | 0.16 |
| 5 | 5.42 | * | 0.29 | 86.81 | ** | 0.76 | 2.92 | * | 0.18 |
| 6 | 5.12 | * | 0.27 | 101.16 | ** | 0.79 | 3.05 | * | 0.18 |
| 7 | 4.63 | * | 0.26 | 109.08 | ** | 0.8 | 2.88 | * | 0.18 |
| 8 | 4.9 | * | 0.27 | 121.1 | ** | 0.82 | 3.04 | * | 0.18 |
| 9 | 4.7 | * | 0.26 | 123.27 | ** | 0.82 | 2.87 | * | 0.18 |
| 10 | 4.72 | * | 0.26 | 130.04 | ** | 0.83 | 2.93 | * | 0.18 |

**Table E. The results of the sensitivity analysis of the aiming angles - comparisons within groups.**

| | | Late BL1 vs. Early TRN | | | Early TRN vs. Late TRN | | | Late BL1 vs. Late TRN | | |
|---|---|---|---|---|---|---|---|---|---|---|
| **Group** | **n** | $t_{27}$ | **p** | **d** | $t_{27}$ | **p** | **d** | $t_{27}$ | **p** | **d** |
| Extrinsic | 1 | 1.69 | 1 | 0.89 | 3.3 | * | 1.41 | 7.46 | ** | 1.88 |
| | 2 | 2.09 | 0.68 | 0.85 | 3.26 | * | 1.26 | 8.57 | ** | 2.19 |
| | 3 | 3.18 | 0.05 | 1.14 | 2.93 | 0.1 | 1.15 | 8.72 | ** | 2.33 |
| | 4 | 4.59 | * | 1.5 | 2.85 | 0.12 | 1 | 9.42 | ** | 2.49 |
| | 5 | 5.43 | * | 1.76 | 2.68 | 0.18 | 0.97 | 9.6 | ** | 2.54 |
| | 6 | 6.11 | ** | 2.01 | 2.23 | 0.51 | 0.98 | 10.19 | ** | 2.68 |
| | 7 | 6.61 | ** | 2.23 | 2.15 | 0.61 | 1 | 10.31 | ** | 2.86 |
| | 8 | 7.1 | ** | 2.37 | 1.71 | 1 | 0.81 | 10.49 | ** | 2.88 |
| | 9 | 7.33 | ** | 2.44 | 1.31 | 1 | 0.65 | 10.74 | ** | 3.02 |
| | 10 | 7.54 | ** | 2.5 | 0.95 | 1 | 0.49 | 10.89 | ** | 3.08 |
| Intrinsic | 1 | 0.24 | 1 | 0.06 | 5.67 | ** | 1.4 | 8.29 | ** | 3.64 |
| | 2 | 0.85 | 1 | 0.19 | 5.43 | ** | 1.18 | 9.43 | ** | 4.02 |
| | 3 | 2.16 | 0.59 | 0.52 | 4.32 | * | 1.02 | 8.92 | ** | 4.34 |
| | 4 | 2.96 | 0.1 | 0.72 | 4.7 | * | 1.17 | 9.22 | ** | 4.25 |
| | 5 | 3.39 | 0.03 | 0.85 | 4.98 | ** | 1.22 | 9.29 | ** | 4.63 |
| | 6 | 3.94 | * | 0.99 | 4.99 | ** | 1.11 | 10.09 | ** | 4.92 |
| | 7 | 4.47 | * | 1.15 | 4.72 | * | 1.07 | 9.98 | ** | 5.13 |
| | 8 | 4.76 | ** | 1.27 | 4.49 | * | 1.03 | 10.03 | ** | 5.42 |
| | 9 | 4.87 | ** | 1.29 | 4.25 | * | 0.98 | 10.19 | ** | 5.6 |
| | 10 | 5.17 | ** | 1.38 | 3.81 | * | 0.93 | 10.42 | ** | 5.7 |
| Control | 1 | 0.64 | 1 | 0.21 | 6 | ** | 2.09 | 7.24 | ** | 2.4 |
| | 2 | 1.59 | 1 | 0.73 | 4.24 | * | 2.83 | 9.05 | ** | 2.98 |
| | 3 | 2.76 | 0.15 | 1.29 | 3.49 | * | 1.48 | 8.78 | ** | 2.56 |
| | 4 | 3.82 | * | 1.92 | 3.45 | * | 1.44 | 9.04 | ** | 2.72 |
| | 5 | 4.32 | * | 2.03 | 3.76 | * | 1.6 | 9.27 | ** | 2.71 |
| | 6 | 4.64 | * | 2.12 | 3.88 | * | 1.77 | 9.9 | ** | 2.95 |
| | 7 | 4.83 | ** | 1.93 | 4 | 0* | 1.64 | 9.77 | ** | 2.69 |
| | 8 | 5.05 | ** | 1.93 | 4.01 | * | 1.55 | 9.98 | ** | 2.73 |
| | 9 | 5.1 | ** | 1.96 | 3.98 | * | 1.48 | 10.24 | ** | 2.74 |
| | 10 | 5.12 | ** | 1.95 | 3.98 | * | 1.3 | 10.52 | ** | 2.79 |

| | | Late BL2 vs. Early TFR | | | Early TFR vs. Late TFR | | | Late BL2 vs. Late TFR | | |
|---|---|---|---|---|---|---|---|---|---|---|
| **Group** | **n** | $\mathbf{t_{27}}$ | **p** | **d** | $\mathbf{t_{27}}$ | **p** | **d** | $\mathbf{t_{27}}$ | **p** | **d** |
| Extrinsic | 1 | 4.19 | * | 1.69 | 3.18 | 0.05 | 1.09 | 0.87 | 1 | 0.23 |
| | 2 | 4.19 | * | 2.02 | 4.02 | * | 1.28 | 0.65 | 1 | 0.21 |
| | 3 | 3.68 | * | 2.78 | 5 | ** | 1.47 | 0.13 | 1 | 0.05 |
| | 4 | 4.26 | * | 2.37 | 4.73 | ** | 1.16 | 0.29 | 1 | 0.11 |
| | 5 | 4.23 | * | 2.23 | 4.39 | * | 1 | 0.56 | 1 | 0.23 |
| | 6 | 4.63 | * | 2.26 | 4.19 | * | 0.91 | 0.54 | 1 | 0.19 |
| | 7 | 4.71 | * | 2.25 | 3.8 | * | 0.84 | 0.52 | 1 | 0.19 |
| | 8 | 4.76 | ** | 2.14 | 3.94 | * | 0.85 | 0.52 | 1 | 0.17 |
| | 9 | 4.26 | * | 1.71 | 3.68 | * | 0.8 | 0.46 | 1 | 0.16 |
| | 10 | 4.5 | * | 1.52 | 3.84 | * | 0.84 | 0.48 | 1 | 0.17 |
| Intrinsic | 1 | 0.22 | 1 | 0.05 | 0.21 | 1 | 0.05 | 0.01 | 1 | 0 |
| | 2 | 0.79 | 1 | 0.24 | 0.39 | 1 | 0.1 | 0.4 | 1 | 0.17 |
| | 3 | 0.73 | 1 | 0.25 | 0.6 | 1 | 0.17 | 0.28 | 1 | 0.13 |
| | 4 | 0.67 | 1 | 0.22 | 0.52 | 1 | 0.18 | 0.21 | 1 | 0.1 |
| | 5 | 0.78 | 1 | 0.29 | 0.43 | 1 | 0.17 | 0.36 | 1 | 0.17 |
| | 6 | 0.79 | 1 | 0.28 | 0.29 | 1 | 0.12 | 0.42 | 1 | 0.17 |
| | 7 | 0.71 | 1 | 0.23 | 0.28 | 1 | 0.12 | 0.31 | 1 | 0.12 |
| | 8 | 0.56 | 1 | 0.18 | 0.24 | 1 | 0.09 | 0.26 | 1 | 0.09 |
| | 9 | 0.63 | 1 | 0.23 | 0.36 | 1 | 0.14 | 0.22 | 1 | 0.08 |
| | 10 | 0.75 | 1 | 0.27 | 0.46 | 1 | 0.19 | 0.23 | 1 | 0.08 |
| Control | 1 | 1.67 | 1 | 0.72 | 0.34 | 1 | 0.15 | 1.39 | 1 | 0.52 |
| | 2 | 2.32 | 0.42 | 0.6 | 0.31 | 1 | 0.12 | 1.79 | 1 | 0.46 |
| | 3 | 2.71 | 0.18 | 0.61 | 0.82 | 1 | 0.36 | 1.94 | 0.94 | 0.44 |
| | 4 | 2.79 | 0.14 | 0.67 | 0.71 | 1 | 0.31 | 1.95 | 0.92 | 0.47 |
| | 5 | 2.96 | 0.1 | 0.67 | 0.59 | 1 | 0.28 | 2.08 | 0.7 | 0.47 |
| | 6 | 3.07 | 0.07 | 0.73 | 0.77 | 1 | 0.44 | 1.9 | 1 | 0.47 |
| | 7 | 3.25 | * | 0.81 | 0.61 | 1 | 0.32 | 1.94 | 0.94 | 0.5 |
| | 8 | 3.33 | * | 0.86 | 0.92 | 1 | 0.68 | 1.92 | 0.99 | 0.56 |
| | 9 | 3.37 | * | 0.84 | 0.8 | 1 | 0.54 | 2.1 | 0.68 | 0.56 |
| | 10 | 3.4 | * | 0.92 | 0.65 | 1 | 0.37 | 2.24 | 0.5 | 0.6 |

**Table F. The results of the sensitivity analysis of the aiming angles - comparisons between groups.**

| | | Extrinsic vs. Intrinsic | | | Extrinsic vs. Control | | | Intrinsic vs. Control | | |
|---|---|---|---|---|---|---|---|---|---|---|
| **Stage** | **n** | $t_{27}$ | **p** | **d** | $t_{27}$ | **p** | **d** | $t_{27}$ | **p** | **d** |
| Early TRN | 1 | 2.17 | 0.12 | 1.01 | 2.51 | 0.06 | 1.28 | 0.34 | 1 | 0.13 |
| | 2 | 1.83 | 0.23 | 0.73 | 1.09 | 0.86 | 0.68 | 0.74 | 1 | 0.3 |
| | 3 | 1.65 | 0.33 | 0.64 | 1.09 | 0.86 | 0.64 | 0.56 | 1 | 0.24 |
| | 4 | 1.99 | 0.17 | 0.78 | 1.17 | 0.75 | 0.64 | 0.82 | 1 | 0.36 |
| | 5 | 2.21 | 0.11 | 0.86 | 1.39 | 0.52 | 0.78 | 0.82 | 1 | 0.36 |
| | 6 | 2.21 | 0.11 | 0.87 | 1.55 | 0.4 | 0.89 | 0.66 | 1 | 0.28 |
| | 7 | 1.99 | 0.17 | 0.8 | 1.64 | 0.34 | 0.9 | 0.35 | 1 | 0.15 |
| | 8 | 2.1 | 0.14 | 0.85 | 1.8 | 0.25 | 0.96 | 0.3 | 1 | 0.13 |
| | 9 | 2.14 | 0.13 | 0.87 | 1.92 | 0.2 | 1.02 | 0.22 | 1 | 0.09 |
| | 10 | 2.03 | 0.16 | 0.82 | 2.01 | 0.16 | 1.07 | 0.02 | 1 | 0.01 |
| Late TRN | 1 | 0.72 | 1 | 0.33 | 0.9 | 1 | 0.35 | 0.18 | 1 | 0.09 |
| | 2 | 0.57 | 1 | 0.26 | 0.75 | 1 | 0.29 | 0.18 | 1 | 0.09 |
| | 3 | 0.82 | 1 | 0.39 | 0.89 | 1 | 0.38 | 0.07 | 1 | 0.03 |
| | 4 | 0.85 | 1 | 0.4 | 0.92 | 1 | 0.38 | 0.07 | 1 | 0.03 |
| | 5 | 0.84 | 1 | 0.39 | 0.82 | 1 | 0.33 | 0.02 | 1 | 0.01 |
| | 6 | 0.7 | 1 | 0.32 | 0.72 | 1 | 0.29 | 0.02 | 1 | 0.01 |
| | 7 | 0.67 | 1 | 0.32 | 0.75 | 1 | 0.3 | 0.08 | 1 | 0.04 |
| | 8 | 0.74 | 1 | 0.34 | 0.69 | 1 | 0.28 | 0.05 | 1 | 0.02 |
| | 9 | 0.79 | 1 | 0.37 | 0.68 | 1 | 0.27 | 0.1 | 1 | 0.05 |
| | 10 | 0.69 | 1 | 0.33 | 0.53 | 1 | 0.21 | 0.16 | 1 | 0.08 |
| Early TFR | 1 | 2.18 | 0.11 | 0.9 | 3.64 | * | 2 | 1.45 | 0.47 | 0.61 |
| | 2 | 2.65 | * | 1.16 | 4.43 | ** | 2.23 | 1.77 | 0.26 | 0.74 |
| | 3 | 3.19 | * | 1.45 | 5.61 | ** | 2.83 | 2.42 | 0.07 | 0.97 |
| | 4 | 3.53 | * | 1.68 | 6.18 | ** | 2.95 | 2.65 | * | 1.06 |
| | 5 | 3.8 | * | 1.79 | 6.55 | ** | 3.08 | 2.75 | * | 1.13 |
| | 6 | 3.79 | * | 1.79 | 6.55 | ** | 2.88 | 2.76 | * | 1.19 |
| | 7 | 3.7 | * | 1.69 | 6.53 | ** | 2.83 | 2.83 | * | 1.28 |
| | 8 | 3.68 | * | 1.7 | 6.5 | ** | 2.77 | 2.82 | * | 1.29 |
| | 9 | 3.28 | * | 1.49 | 6.04 | ** | 2.52 | 2.75 | * | 1.31 |
| | 10 | 3.26 | * | 1.44 | 5.96 | ** | 2.45 | 2.7 | * | 1.36 |
