# Supplementary material for: Orientation control strategies and adaptation to a visuomotor perturbation in rotational hand movements
Source: PLoS Comput Biol. 2022 Dec 5;18(12):e1010248. doi: 10.1371/journal.pcbi.1010248 (PMC9754612; doi:10.1371/journal.pcbi.1010248)
Supplement: Attachment — Submitted filename: point_by_poiny_PLOS.pdf [file pcbi.1010248.s002.pdf]

Dear editor,

We thank you and the reviewers for thorough feedback on our manuscript. We took all the comments on board and changed the manuscript substantially to address these important concerns. The main changes we made are:

1. We clarified the task settings of experiment 1.
2. We analyzed the learning of the QGS values in experiment 1.
3. We focus more on presenting the methodology and its demonstration using two experiments, rather than on the conclusions.
4. We base our inferences on effect sizes and not only on p-values.
5. We performed a QGS analysis on the baseline trials in experiment 2 to support the reliability of the adaptation session.
6. We conducted a sensitivity analysis on the number of averaged trials in experiment 2, reported the three-trials analysis, and provided the results of all analyses in the appendices.
7. We added several figures and panels (Fig. 2d, Fig. 7, Fig. 9, Fig. 10, and Fig. 11d).
8. We discuss the use of quaternions in representing orientation compared to other representations.

The specific changes to the manuscript are presented in the detailed answers to the comments of the reviewers. Throughout the text, we use the following color-coding:

- Original comments by the reviewers – black regular font
- Our responses – *blue italic font*
- Citation from the paper – **“red bold font in quotation marks”**

We have also attached a document that highlights the changes in the manuscript (removed words are crossed out and colored red, and added words are colored blue), and a clean version of the new manuscript.

Thank you for your consideration.

Reviewer #1

Here the authors use a new experimental paradigm to examine how the sensorimotor system adapts to visual rotations that have been perturbed about a 3D axis. The authors use quaternions to perform the kinematic analyses, which have benefits over more common and traditionally used affine transformation approaches (Euler, Cardan, helical axis). The authors cleverly exploit that quaternion multiplication is not commutative to get at the notion that humans adapt in extrinsic verses intrinsic coordinate frames. Overall, I am generally positive as there is merit to their new approach, which is largely a methods paper and differs from the commonly used visuomotor rotation paradigm. Comments, questions, and concerns are mentioned below. Can the authors please respond in a point-by-point manner.

1. For the extrinsic and intrinsic groups in Experiment 2, the two groups rotated about two very different axes and performed very different movement patterns during training. A concern here is that a history of being reinforced along very different axes of rotation and movement patterns may have had different carry-over effects into the transfer task, irrespective of learning an intrinsic or extrinsic coordinate system. That is, given how different the rotations / movements were during training, this would seem like a potential confound when comparing groups in the transfer task. Further, in both groups participants must adapt the intrinsic coordinates to carry out the task, since the upper arm was left completely unconstrained. Since this is a new technique and the authors' suggested main finding hinges on two conditions with very different movement patterns during training, it would have been useful to test many more axis rotations to be assured that the results are not merely a coincidence but a robust finding. Can the authors please comment and include any changes to the text that might bolster their suggestion.

Thank you for this important comment. Indeed, there is a possibility that the transfer of adaptation in the Extrinsic group was affected by the specific trained target, regardless of the trained coordinates. The aim of this article is to introduce new methodologies when analyzing orientation trajectories, and demonstrate these methodologies in two experiments. As such, we introduced an analysis, an experimental setup, and a visuomotor rotation that remaps the orientation of the hand, rather than its position, and we demonstrate them with results "akin" to classical experimental findings about the straight path and bell-shaped speed trajectory of reach movements, and about adaptation to visuomotor rotation in planar cursor movements. To this end, we now highlight that the emphasis of our paper is the focus on the methodologies, rather than on the conclusion about the coordinate system. Future work is needed to establish a more thorough understanding of the adaptation and the generalization of this perturbation, possibly by testing many different targets and initial orientation. We refer to this limitation in the procedure and protocol section:

"We chose the target orientations such that the ideal rotation axes (i.e., the axes used when following a geodesic) would be orthogonal between the two groups in BL1 and TRN, but identical in BL2 and TFR. Therefore, a difference between groups in the transfer of adaptation to an orthogonal initial orientation could be associated with the coordinates in which the perturbation was learned. However, such difference may also stem from the

difference in the training targets, regardless of the trained coordinates (see the Discussion section).”

We also discuss this limitation in the discussion:

“To test in which coordinate system the visuomotor rotation was represented, we chose the ideal rotation axes to be orthogonal between the Extrinsic group and the Intrinsic group during training. As a result, participants in these two groups rotated towards different targets. This raises the question of whether the trained target had an effect when transferring to a new initial orientation. That is, it may be that the transfer of adaptation that we observed in the Extrinsic group stems from the specific training target, regardless of the trained coordinates. Nevertheless, our study first and foremost established a new viosuomotor rotation methodology. Furthermore, our results need to be further validated with many more rotation axes, initial hand orientation, and targets to ascertain the robustness of our finding [25].”

To emphasize our methodologies, in addition to changing the text, we created a graph that visualizes the comparisons that we made during the spherical axis analysis (Fig. 9, see comment 2c). Using this grapgh, the reader can see the differences in aiming axes between different stages within and between groups.

2. The authors use only one data point (e.g., trial 301) for several of the comparisons, which is likely quite suspectable to noise. The adaptation does occur quite quickly, so perhaps taking the median of three trails for all the points of interest (early learning, late learning, early transfer, etc.) to create more robust statistics. This is highlighted when the authors do not have statistical significance between the extrinsic verse intrinsic groups (lines 651-656) with a single trial analysis, which is critical to their suggestion that humans use an extrinsic coordinate system (global) as opposed to an intrinsic coordinate system (e.g., joint or muscle space) to adapt to the perturbed 3D rotations. It is not sufficient that there are within participant differences between training and transfer, as currently shown in the Figure 8, to make this suggestion. That said, significance ($p < 0.05$) is an arbitrary line in the sand. Further, the authors do find significance when considering three trials.

2a. Instead, the authors should focus on reporting effect sizes (also reported on the figures).

We agree with the reviewer, and shifted focus from p-values to effect sizes. In the spherical aiming axes analysis, the effect size is reported using the mean of the angular distance between the compared groups, as it is easy to interpret. This is mentioned in the statistical analysis section:

“In the spherical aiming axes analysis, the effect size is reported using the mean of the angular distance between the compared groups, as it is easy to interperet.”

The effect sizes are reported in the text (results section), as well as in the figures (Fig. 9 and Fig. 11).

In the scalar aiming angle analysis, the effect size of the ANOVA is reported using partial squared η, and of the multiple comparisons using Cohen's d:

"The effect size is reported using partial eta-squared (η_p^2) for the ANOVA test and Cohen's d for all t tests."

2b. Perform a sensitivity analysis using different numbers of trials (e.g., 1-10 trials).

We performed a sensitivity analysis on the number of trials averaged in each key stage (late baseline 1, late baseline 2, early training, late training, early transfer, and late transfer). We repeated the analysis with all possible options between averaging over 1 to 10 trials. The full results of this sensitivity analysis is reported in the supporting information (aiming axes sensitivity analysis in Tables 2-4, and aiming angles sensitivity analysis in Tables 5-7):

We chose to report in the text the three-trials analysis, as it was a fair compromise between not missing the fast learning and not relying on a noisy single-trial analysis. Of course, a single-trial analysis would be better in capturing fast processes, such as the learning we observed, yet, as mentioned, it is prone to noise. This tradeoff is specifically mentioned in the statistical analysis section:

"A visual examination of the adaptation curves implied that the learning was fast, but the transfer was slow. Fast processes are easier to observe when using a single-trial analysis (i.e., considering only the first trial of TRN as representing the effect of the initial exposure to the perturbation) instead of a multiple-trials analysis (i.e., using the first few trials of TRN to characterize the effect of the initial exposure). Slow processes, on the other hand, are still visible when using multiple-trials analysis. However, using a single trial-analysis is prone to be noisy, as opposed to multiple-trials analysis. To be able to observe both fast and slow processes, we conducted a sensitivity analysis on the number of trials averaged in the statistical analysis, i.e., we compared within and between groups each time by averaging over a different number of trials (1-10). For example, when using three trials in the analysis, we averaged over trials 118-120 (late BL1), as well as trials 178-180 (late BL2), trials 181-183 (early TRN), trials 358-360 (late TRN), trials 361-363 (early TFR), and trials 418-420 (late TFR). We report the statistical results of a three-trials analysis, as it is able to capture large effects of both learning and transfer behaviour. However, we provide all the results from the sensitivity analysis in S1 Appendix A (aiming axes analysis) and S2 Appendix B (aiming angles analysis)."

We briefly mention the limitation of using the three-trials analysis when studying fast learning curves in the results section:

"Moreover, averaging over three axes is expected to result in large difference between the late BL1 stage and the early TRN stage if the learning is fast. In all groups, we found a large difference (Extrinsic: χ_2^2 = 37.06, p < 0.001, mean diff. = 22.5°, Intrinsic: χ_2^2 = 5.54, p = 0.06, mean diff. = 15.1°, Control: χ_2^2= 3.86, p < 0.001, mean diff. = 19.4°). However, this is likely due to the fast learning in the Intrinsic and Control groups, as a single-trial analysis revealed only small differences (Intrinsic: χ_2^2= 1.3, p = 0.52, mean diff. = 9.4°, Control: χ_2^2= 1.2, p = 0.55, mean diff. = 7.1°)."

The tradeoff is also discussed in the discussion section:

“Both the spherical aiming axes analysis and the scalar aiming angles analysis suggested that the adaptation of the rotation-based visuomotor rotation took place within extrinsic coordinates. This is suggested from the results of the three-trials analysis, in which participants in the Extrinsic group showed a large aftereffect in the transfer of the adaptation to a new initial orientation, while participants in the Intrinsic group and the Control group did not. Along with the large difference between the Extrinsic group and the Intrinsic group in the early TFR stage, this result supports the extrinsic learning hypothesis (see Fig. 4). We chose to report the results of the three-trials analysis as it nicely captured both the learning and transfer effects, and is less prone to noise than a single-trial analysis. However, while this choice is compatible with the slow washout observed in the Extrinsic group, it may underestimate the fast learning in the early TRN stage of all groups. Nevertheless, even the single-trial analysis was able to capture a large difference between the Extrinsic group and the Intrinsic group in the early TFR stage, though this difference was not statistically significant. The choice of the number of trials to average is somewhat arbitrary, therefore, we provided the full results of the sensitivity analysis for both the spherical aiming axes analysis (see S1 Appendix A) and the scalar aiming angles analysis (see S2 Appendix B).”

In addition to reporting the three-trials analysis in the text, we also use it in the figures of the spherical aiming axis comparisons (Fig. 9), of the velocities at key stages (Fig. 10), and of the scalar aiming angles comparisons (Fig 11.c-d). That it, the aiming axes, velocity profiles and aiming angles that are plotted are averaged over three trials.

2c. They should also include a figure (e.g., 8D) that shows the group comparisons (intrinsic vs extrinsic vs control) for the transfer portion.

We added panel (d) in Fig. 11 (former Fig. 8) to show the group comparisons of the aiming angles in the early training stage, in the late training stage, and in the early transfer stage. These comparisons show the lack of large differences between groups in the early and late TRN trials, as well as a large difference in the early TFR stage:

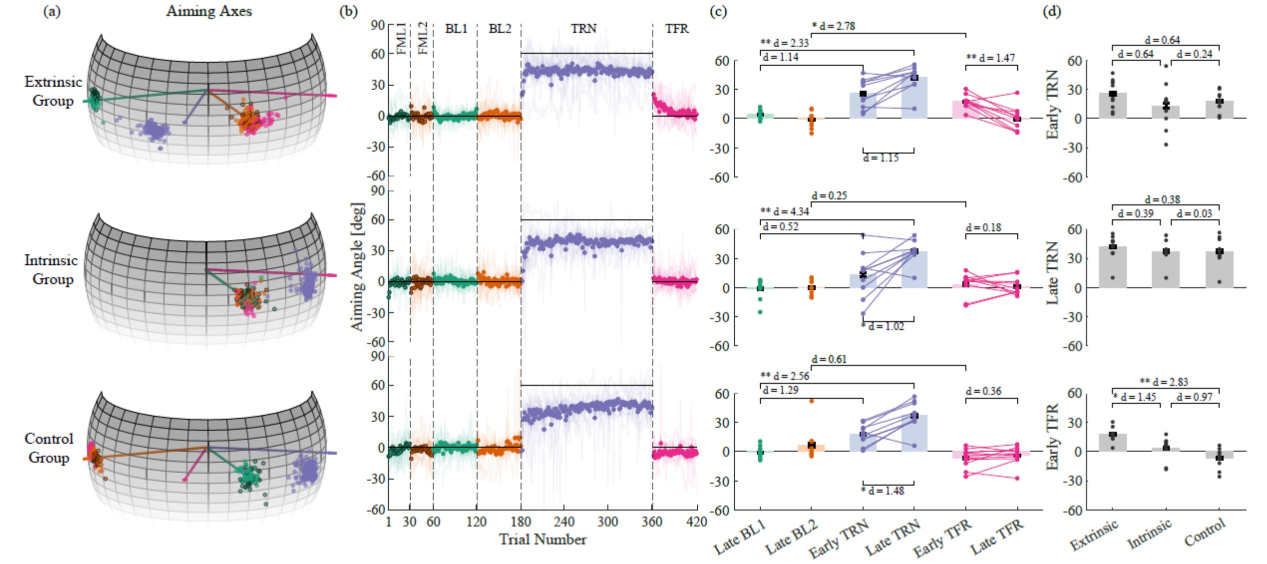

Fig 11. Average aiming axes and angles of all groups in experiment 2 and comparisons of the aiming angles between and within groups. The average aiming axes and angles are consistent with a representation of the partially learned perturbation in extrinsic coordinates and with a narrow generalization to different targets. The colors are as in Fig. 8. (a) The course of the aiming axes (dots) averaged over participants in each group, and the ideal axis (arrows) in each session. (b) The time course of the aiming angles (dots) averaged over participants and the standard error (dark shading) of the aiming angles in each group. The light curves show the time courses of the aiming angles of each participant. The dashed lines separate between the different sets of trials. (c) Within groups comparisons of the three-trials averaged aiming angles of all participants (dots) in each of the key stages. The bars and error bars are the mean ± standard error. *p < 0.05, **p < 0.001. The effect sizes are in the inset. (d) Between groups comparisons of the three-trials averaged aiming angles of all participants in the early TRN stage, the late TRN stage, and the early TFR stage).

We refer to Fig. 11d when presenting the between group differences found using the aiming angles analysis (see Results section):

"The groups differed only in the early TFR trials (Fig. 11d, Extrinsic vs. Intrinsic: t_{27} = 3.19, p < 0.05, d = 1.45, Extrinsic vs. Control: t_{27} = 5.61, p < 0.001, d = 2.83, Intrinsic vs. Control: t_{27} = 2.42, p = 0.07, d = 0.97). The large difference between the Intrinsic group and the Control group is due to the negative deviation of participants in the Control group during the early TFR stage. All the other comparisons between groups in the three-trials analysis yielded non-significant and small differences (see Table 7)."

We also added Fig. 9 (see below) that shows the within group comparisons between key stages (a-f) and the between group comparisons within key stages (g). The figure visualizes the comparisons and show the results discussed in the text, among them the partial adaptation in all groups, and the partial transfer in the Extrinsic group. We refer to Fig. 9 throughout the text of the results of experiment 2, as well as in the caption of Fig 9.

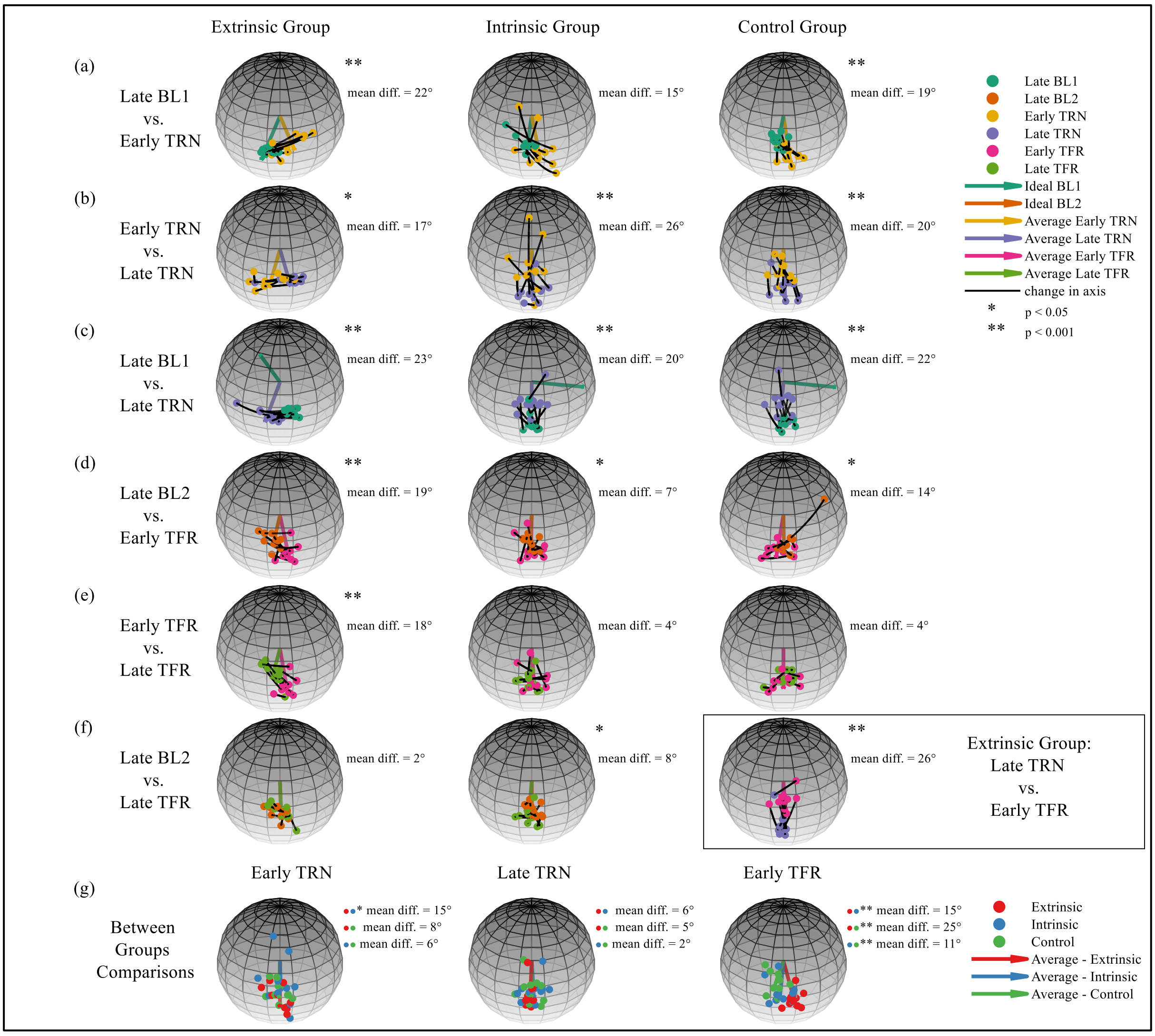

Fig 9. Within and between groups spherical analysis. The spheres show the comparisons of the three-trials analysis within groups between key stages (a)-(f), and between groups within key stages (g). The dots show the average axis of each participant, and the arrows depict either the mean of the sample or the ideal sample (see legend). The view of each sphere was adjusted for visibility. The within group analysis shows an effect of the initial exposure to the perturbation which may stem from correction attempts or from using the three-trials analysis (a). Furthermore, it shows a clear adaptation throughout the TRN session (b)-(c) and a transfer of adaptation only in the Extrinsic group (d), which is washed out by the end of the TFR session (e)-(f, left and middle). However, the transfer is only partial (f, right). The between group analysis shows a large difference in the initial exposure to the perturbation between the Extrinsic group and both the Intrinsic and control groups (g, left). It also shows no difference in the late TRN axes, indicating similar adaptation patterns (g, middle). Most importantly, it highlights the difference in TFR between the Extrinsic and Intrinsic groups (g, right).

2d. Further, they should include text in the discussion that highlights this limitation.

We performed a full sensitivity analisys and now report average over 3 trials. We added a discussion about the tradeoff in analysis choices (see comment 2b), and also the limitation of using p-values and the importance of relying on effect sizes together with the significance:

"Furthermore, we report differences within and between groups using effect sizes, rather than just p-values, since a significant difference is not necessarily a large difference."

2e. In this case, it would be useful to including the supplementary in the main text since the two metrics support one another.

All the analyses are reported in the main text. The supporting information now includes only the full results of the sensitivity analyses (aiming axes sensitivity analysis in Tables 2-4, and aiming angles sensitivity analysis in Tables 5-7).

3. The manuscript would benefit from plotting more dependent measures. The authors do report overall adaptation angle on a trial-by-trial level. It would be useful to plot the angles/velocity over time for each group, during key trials (e.g., early adaptation). Perhaps this is difficult with the instantaneous axis, but one could conceivably use the average axis. The authors do report individual angular velocities along each DoF, but it would be useful to also plot their Euclidean norm.

In the results of experiment 1, we added the norm of the angular velocity to figure 5 (middle column, black curve):

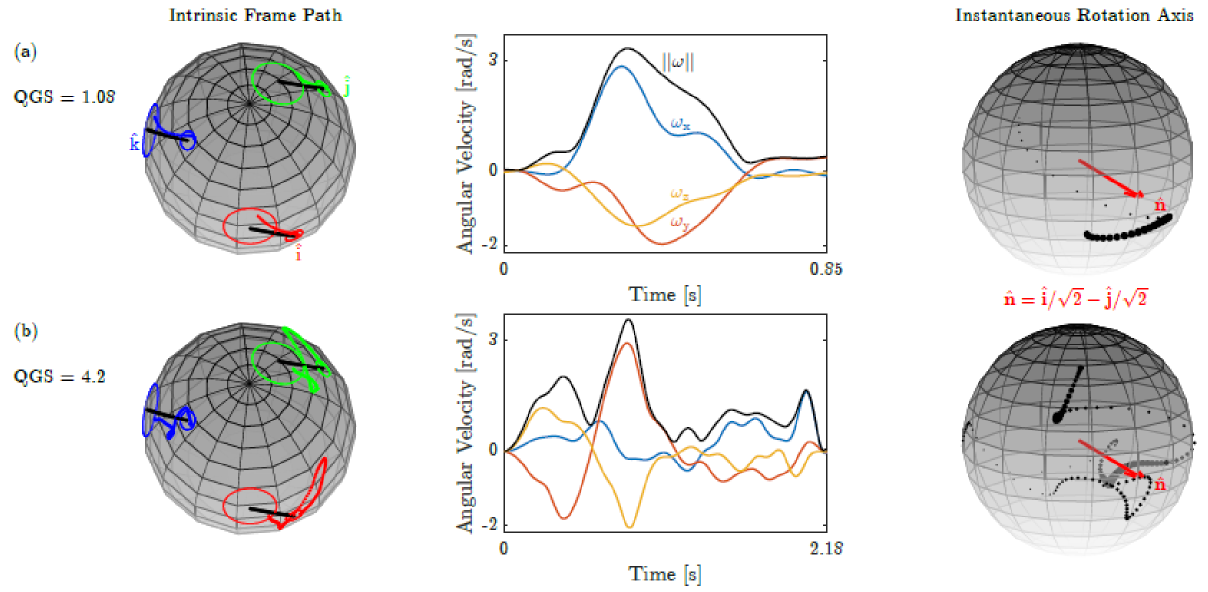

Fig 5. Examples of a geodesic and a non-geodesic. The left column shows the path of the intrinsic reference frame that is attached to the robotic handle ($\hat{i}$ - red, $\hat{j}$ - green and $\hat{k}$ - blue). The circles enclose caps that define the accepted range for the hand's orientation at the initiation of the movement (small cap with angular aperture of 5°) and at its termination (large cap with angular aperture of 15°). The black curves describe the geodesic between the initial cursor orientation and the target orientation. The middle column shows the angular velocity profile (blue, orange, and yellow curves) used to follow the orientation path, as well as its norm (black curve), and the right column describes the path of the instantaneous rotation axis (black dots). To emphasize fast segments, the size of the black dots is scaled in proportion to the angular speed. The ideal rotation axis is shown in red. (a) The trajectory of the intrinsic frame of a geodesic follows a short path on the sphere and is characterized by a smooth angular velocity profile and a concentrated instantaneous axis path. (b) The trajectory of the intrinsic frame of a non-geodesic follows a longer path on the sphere compared to that of the geodesic and is characterized by a fragmented angular velocity profile and a dispersed instantaneous axis path. In both examples, the initial cursor orientations and the target orientations were identical.

In the results of experiment 2, we added Fig. 10, which shows the three-trials averaged velocity profile of the participants in each group and in each key stage of the experiment. We normalized the time to be able to show an average over the velocity profiles. We also normalized the angular velocity of each participant to make its norm (i.e., the scalar speed) to be between 0 and 1. The trajectory of the norm of the velocity is also shown. This figure

complements the existing figures. To be compatible with the rest of the figures, the velocities are shown within an intrinsic reference frame. From examining this figure, it is evident that the adaptation exists in all groups, but the transfer exists only in the Extrinsic group.

We present the figure in the results section:

"To complement the spatial spherical analysis with spatio-temporal characteristics of the rigid body's rotation, we present the intrinsically represented three-trials averaged, angular velocities of each participants in each key stage in Fig. 10. To make the comparison feasible, we normalized the time to be between 0 and 1, as well as normalized the angular velocity of each participant, such that the scalar speed (i.e., the norm of the angular speed) is between 0 and 1."

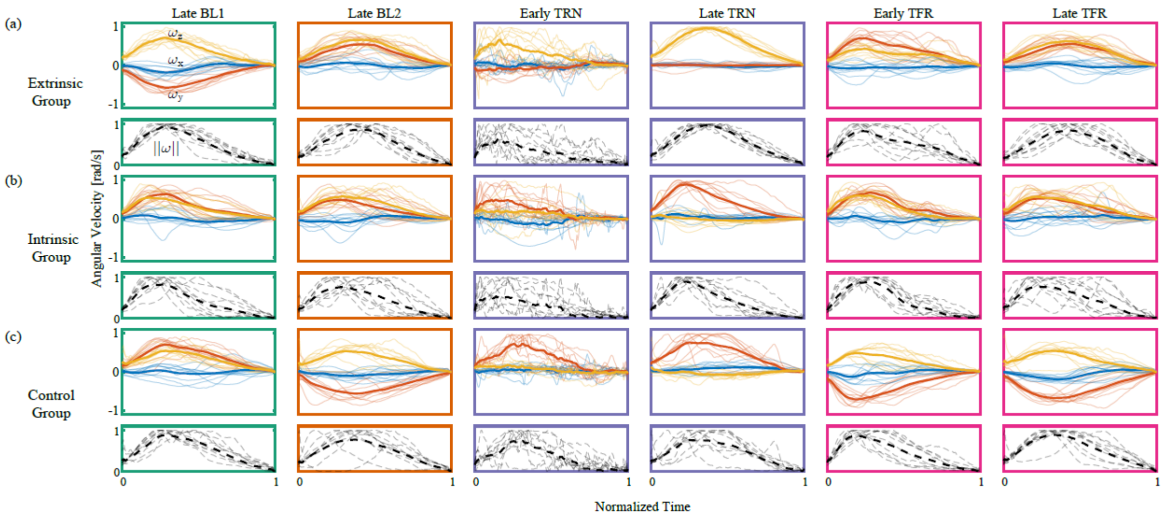

Fig 10. Comparison of angular velocity profiles at key stages. The solid shaded curves depict the three-trials averaged, time and amplitude normalized, angular velocity profile of each participant in key stages in intrinsic coordinates (x in blue, y in orange, and z in yellow). The solid bold curves depict the time and amplitude normalized angular velocity profile averaged across participants. The dashed gray and black curves depict the individual and normalized angular speed, respectively. (a) Extrinsic group, (b) Intrinsic group, and (c) Control group. In each group, the upper panel depicts the velocity and the lower panel depicts the scalar speed. The velocity analysis complements the spherical axis analysis, as the ratio of between the coordinates of the velocity profiles (i.e., the normalized vector) is the aiming axis.

These observations are reported in the results section along with the results of the aiming axes analysis. First, they inform on the effect of initial exposure to the perturbation:

"The three-trials averaged, time and amplitude normalized, angular velocities clearly reveals the difference in the aiming axes, as the ratio of the x, y, and z coordinates differs between the late BL1 trials and the early TRN trials in all groups. They also show a fragmented rotation compared to the smooth bell-shaped velocity profile in the late BL1 trials."

Then, the ratio between the components of the angular velocity, which is the rotation axis, reveals the partial adaptation:

In fact, by the end of the TRN session, participants in the Extrinsic group rotated on average around an axis near the flexion-extension axis (k, Fig 10a, Late TRN), while

participants in the Intrinsic and Control groups rotated on average around an axis near the radial-ulnar axis (j, Fig. 10b, Late TRN).”

Furthermore, the angular velocities show the transfer of adaptation in the Extrinsic group:

“The transfer of adaptation in the Extrinsic group is also evident from the change in velocity coordinates between late BL2 trials and early TFR trials (Fig. 10a), as opposed to a similar velocity profile in the same stages of participants in the Intrinsic group (Fig. 10b). This result indicates that, on average, the adaptation was represented in extrinsic, rather than intrinsic, coordinates.”

Last, the angular velocity profiles show that participants regained baseline behavior by the end of the transfer session:

“In fact, they regained BL2 behaviour, as indicated by the comparison of the aiming axes in late BL2 trials and late TFR trials (Fig. 9f (left), χ_2^2 = 0.47, p = 0.79, mean diff. = 2.4°), and by the comparison of the angular velocity profiles in these stages (Fig. 10a).”

Another useful metric here would be movement times.

The trial's duration is indeed an important metric when studying adaptation in motor control tasks. However, in order to guide the participants to perform their movements within a desired duration range and to reduce variability in the data, we displayed feedback regarding the duration of the movement. We displayed a "Move Slower" notification if the movement lasted less than 400 m] and a "Move faster" notification if it lasted more than 600 ms. Therefore, the trial's duration would not be very informative in our analysis, and we chose not to include it in the manuscript.

Nevertheless, we did the analysis and provide a summary of the analysis below. The trial's duration is within the desired range in most of the first and second baseline trials (the range is depicted by the black horizontal lines). When the perturbation is introduced, participants take longer to perform the trial (higher values in the beginning of the TRN session), possibly since we provided a countinues visual feedback which caused them to attempt to correct their movement. Though, the curves show fast reduction in the trial's duration and convergense to the desired range. Furthermore, the duration does not change when the perturbation is removed.

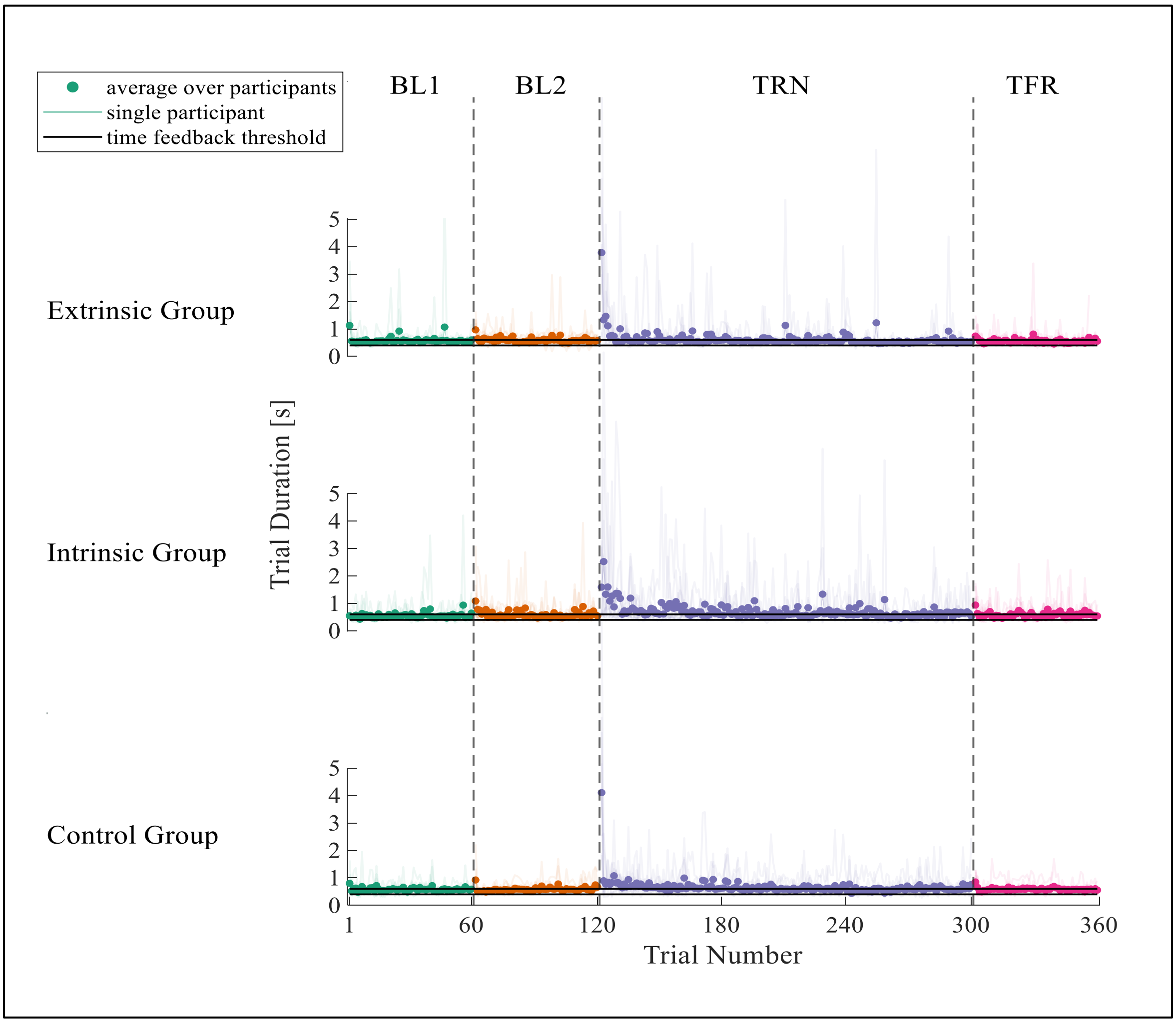

4. The authors should highlight the advantages of quaternions over traditional techniques (no gimbal lock / singularities and less impacted to noise), which the authors should highlight. The obvious downside of quaternions is that they are much harder to interpret and visualize compared to other common techniques, such as Euler, Cardan, Helical axis. Of these, quaternions are like helical axis in spirit, which are much easier to visualize and have been used on the biomechanics literature in the past (Woltring, 1984). Perhaps it is worthwhile to briefly discuss the advantages and disadvantages to their approach.

We added a discussion on the advantages and disadvantages of using quaternions with respect to other possible representations:

"Our computational analysis design is based on the assumption that the nervous system represents the orientation of three-dimensional rigid bodies as quaternions. Quaternions are advantageous compared to other representations as their conversion from rotation matrices is nearly unambiguous. However, each rotation can be converted into two quaternions (q and -q). They also allow to define geodesics (see the Mathematical formulation section). However, quaternions may seem less intuitive compared to other representation, and are hard to visualize, as they are four-dimensional objects. Other representations of orientation include the helical axis representation which describes a moving rigid body as translating and rotating around an axis. Yet, helical axes are more sensitive to measurement errors than quaternions [50]. Euler angles are a parameterization of the rotational space using a series of rotations about three mutually orthogonal axes. Euler angles representation suffers from several disadvantages, among them is the ambiguity in conversion from a rotation matrix to a set of Euler angles, which impose challenges on defining metrics [51], and therefore geodesics. In addition, Euler angles suffer from Gimbal lock - the loss of one rotational degree of freedom when two rotations act about the same axis. The current experiment does not allow for differentiating between different representations of orientation in the nervous system. We chose the quaternions because they provide a convenient computational framework that facilitates further studies, as we showed in the second experiment."

5. A strength of the paper is that the authors have made a new paradigm to study visuomotor rotations. As such, they have derived a nice metric to define the path traversed by the quaternion vector relative to the geodesic. The authors define the geodesics metric, which is quite nice. Perhaps another useful metric to construct is the integral of the deviation from the geodesic, normalized by time.

The integral over the deviation from a geodesic was used in studying of translational reaching movement. This metric quantifies the area in the plane between the straight line and the curved movement. In quaternion space, such metric would require complicated differential geometry. Specifically, it would require to define an orthogonal curve from a sample on the four-dimensional sphere and the geodesic. Then, using methods similar to trapezoidal integration, the equivalent of an area on the four-dimensional sphere could be achieved. We expect such metric to correlate with the QGS that we defined, as they both characterize the geodicity of the rotations. If dynamic perturbations were to be used, the

integral metric could provide additional information on the direction of the deviation from geodicity and be used to infere on the control strategies used. However, we only considered orientation matching without dynamical perturbations, and therefore, we do not think that this metric would provide additional information. For this reason, as well as due to the complexity of the math, we chose not to pursue this specific direction in this study. However, we addressed this point in the discussion:

"In this study we employed a visuomotor rotation for studying adaptation in object manipulation. Classical studies of translational movement employed other types of perturbations, including adaptation to dynamical perturbations, such as viscous force fields [7]. Quaternion-based metrics can also be used in the analysis of rotational hand movements in the presence of dynamical perturbations, such as torque fields. Under such interference, the hand is expected to largely deviate from the geodetic quaternion path. One way to measure such deviation would be to integrate over the quaternion path by defining a curve between each quaternion sample and the geodesic, which intersects with the latter orthogonally. Then, using an interpolation method similar to trapezoidal integration, the integral may be computed. To account for the variability in the trial's duration, the integral may also be time-normalized."

6. Was the time derivative taken on the I,j,k, components to calculate angular velocity?

The angular velocity was computed using Eq. 18: $\omega = \frac{\theta^{\delta}}{\Delta t}\hat{\mathbf{n}}^{\delta}$. θ^{δ} *is the instantaneous angle of rotation, and* $\hat{\mathbf{n}}^{\delta}$ *is the instantaneous axis of rotation (see the mathematical formulation section).* Δt *is the time sampling interval. The components (i,j,and k) come from the axis. We added this equation in the data analysis section.*

7. abstract: "We found that rotations are generally performed by following a geodesic in the quaternion hypersphere, which suggests that, similarly to translation, the orientation of the hand is centrally controlled." It would be useful to have a follow-up sentence here, making the link to why the shortest path indicates central control.

We modified the abstract to make the link to why a geodesic indicates central control:

"In the first experiment, participants performed a simple orientation-matching task with a robotic manipulator. We found that rotations are generally performed by following a geodesic in the quaternion hypersphere, which suggests that, similarly to translation, the orientation of the hand is centrally controlled, possibly by optimizing geometrical properties of the hand's rotation."

8. lines: 10-14: there are several non-kinematic hypotheses that explain this 'invariant' (which does alter shape depending on the task). For example, min torque rate (Uno), OFC, where it arises by minimizing effort costs in the presence of signal dependent noise, as well as energetics (Wong, Kuo, 2021). Perhaps change the language here slightly to be more encompassing.

We added to the introduction a short review of the non-kinematic hypotheses:

"One such invariant is the straight path and bell-shaped velocity trajectory that characterize fast point-to-point movement; these may result from the optimization of movement smoothness [1-4]. This invariant may also suggest that point-to-point hand movements are primarily under kinematic control in extrinsic coordinates that minimizes reaching errors, consistent with models of deterministic optimal control [1]. This hypothesis is reinforced by the robustness of the invariant to visual [5, 6] and dynamical [7, 8] distortions, and the adaptation in the presence of these distortions is explained with state-space models [9-12]. Alternative non-kinematic hypotheses were also suggested to predict the invariant point-to-point arm movements. Such are models of stochastic optimal feedback control [13-15], which minimize efforts in the presence of signal dependent noise, and the minimum torque change model [16], which accounts for the dynamics of the arm. A recent study presented a model that explains both smoothness and duration of reaching movements in terms of economy by considering the expenditure of metabolic energy [17]. Other studies claimed that control strategies also depend on the cognitive perception of the task. For example, straight paths in joint space were observed when a two-link arm was controlled, rather than just the endpoint position [18]."

9. Useful to include the following citation, which examine sensorimotor control using quaternions: Leclercq, G., Lefèvre, P., & Blohm, G. (2013). 3D kinematics using dual quaternions: theory and applications in neuroscience. Frontiers in behavioral neuroscience, 7, 7.

This is a great reference which we previously missed. We now refer to it in the introduction, where we talk about previous studies that investigate the human motor control of the orientation of rigid bodies:

"A theoretical study exploited dual quaternions - a mathematical object used to represent movement in six degrees of freedom – to describe three-dimensional kinematics, and presented its use in models predicting the motion of the hand [45]."

10. Do the instantaneous axes always intersect at the same point?

Yes, the instantaneous axes always intersect at the same point, which is the center of mass of the controlled object (also the origin of the extrinsic and intrinsic coordinate systems, and the center of the virtual cube). We added a clarification in the mathematical formulation section:

"The instantaneous rotation axes intersect in the origin of the coordinate system (the center of mass of the controlled body)"

11. line 335: Include baseline trials. It is always useful to see participants initial behaviour.

As you suggested, we also analyzed the familirization trials where the particpants where first introduced with the task. The trials sets are introduced in the procedure and protocol section:

"To familiarizeparticipants with the orientation-matching task, they performed 60 familiarization trials without any perturbation. Participants performed the first 30 trials (FML1) starting from the unrotated state of the hand ($o_1 = q_I$). In the next 30 trials (FML2), we rotated the initial orientation by 90 around ˆi ($o_2 = 1/\sqrt{2} + \hat{\imath}/\sqrt{2}$)."

This analysis of the aiming axes and angles of the familirization trials was added in Fig. 8a-b (shown below) and in Fig. 11a-b (see figure in comment 2c):

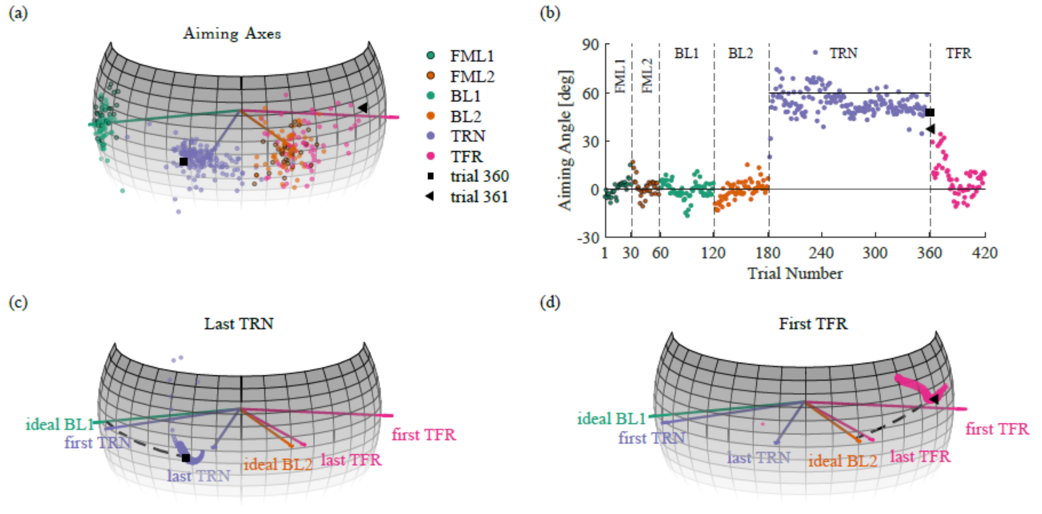

Fig 8. Aiming axes and angles of a single participant in the Extrinsic group. The colors are as in Fig. 3. The FML1 trials are colored as BL1 trials and enclosed by black circles, and the FML2 trials are colored as BL2 trials and enclosed by black circles. (a) The course of the aiming axes. The last TRN axis and the first TFR axis are marked as a square and a triangle, respectively. (b) The time course of the aiming angles. The dashed lines separate between the different sets of trials, and the continuous lines indicate the ideal aiming angle at the BL sessions and the aiming angle required for full adaptation at the TRN session. (c) The rotation axes of the quaternion path in the last TRN trial. The extent of adaptation is the length of the arc that connects between the ideal BL1 axis and the aiming axis (i.e., the quaternion axis at the time of maximal angular speed, square). The arc reaches near the axis that represents full adaptation, which indicates that the participant had largely adapted. (d) The rotation axes of the quaternion path in the first TFR trial. The extent of transfer is the length of the arc that connects between the ideal BL2 axis and the aiming axis (triangle). The arc reaches near the axis that represents full transfer, which indicates that the participant had fully transferred the adaptation to a new initial orientation. To emphasize fast rotation samples, the size of the dots is scaled in proportion to the angular speed in (c) and (d).

12. It will be very important to provide the code, since using quaternions is quite involved.

The code is provided at: https://github.com/Bio-Medical-Robotics-BGU/Orientaiton-control-strategies. *We added this URL in the statistical analysis section.*

Reviewer #2

Many previous studies have focused on the planar reaching movement to investigate problems such as how the motor system plans the trajectory or adapts the movement to a novel environment. The present study has challenged examining hand orientation control, which is important yet not extensively studied. To this end, the authors developed a novel robotic device and VE for the hand orientation-matching task and adopted quaternion-based analysis to evaluate the kinematics.

Exp.1 demonstrated that the QGS value was close to the optimal value in most cases, suggesting that the hand orientation was controlled efficiently like a straight movement path observed in the reaching movement. The authors also tried to impose visual rotation perturbation to this task and based on the generalization pattern after the adaptation, they concluded that the adaptation was likely to be achieved in the extrinsic coordinates.

This is a very interesting study tackling the problem of hand orientation control by using a novel experimental paradigm. This methods will enable to investigate a lot of interesting research
I also felt that adopting quaternion-based data analysis and the task design (initial and target orientation etc.) was very smart. However, I was concerned with several points, particularly about the readability of the paper. Below, I would like to raise several issues that the authors need to consider.

Major points:

1. It was too difficult to understand the task settings. There are several unclear or confusing points. My interpretation of Experiment 1 is as follows, but I am unsure if it is correct.

There are two initial orientations and two target orientations (Table 1). When the initial orientation was given (o1 or o2 condition), the target orientation was determined by rotating the initial orientation around the axes (e.g., \hat{i} or -\hat{j}, etc. in Table 1) by \alpha (40~60 deg). There are also two ways of rotation (intrinsic and extrinsic described in Eqs. (3) and (4)). In contrast, when the target orientation was given (t1 or t2 condition), the initial orientation was determined by inversely rotating the target orientation.

Anyway, the task settings should be described more clearly. Otherwise, I believe that almost all readers should be confused about the task.

Thank you for pointing out the lack of clarity in our description of the task. You understood the settings correctly. We modified the text that presents the task settings in experiment 1.

In the procedure and protocol section, the general structure of the task conditions, as well as the idea behind the choice of initial and target orientations, are explained::

"Each orientation-matching trial was defined by an initial orientation and a target orientation. To test the effect of the initial orientation of the hand on the geodicity of the path, we chose the initial orientation of the hand to be one of two orientations in half of

the trials (o_1 and o_2, Table 1). Similarly, to test the effect of the target orientation, we chose it to be one of two orientations in the other half of the trials (t_1 and t_2, Table 1). When we chose the initial orientation, the target orientation was determined by rotating the initial orientation around an axis (Table 1) by an angle α. When we chose the target orientation, the initial orientation was determined by inversely rotating the target orientation around an axis by an angle α. In addition, we rotated the cursor in two different ways; by rotating with respect to an extrinsic coordinate system (using Eq. 3) and with respect to an intrinsic coordinate system (using Eq. 4)."

The choise of rotation axes is also explained:

"For a given initial orientation, we chose rotation axes that would allow us to test for the effect of the alignment of the ideal rotation axis (i.e., the axis that one should rotate around to move along a geodesic) on the QGS. Therefore, we chose rotation axes that were aligned or misaligned with either an extrinsic or an intrinsic reference frame (see Table 1 and Fig. 2d for examples of the experimental conditions)."

We also added panel (d) to Fig. 2 (shown below) to visualize the task setting better. We provide examples of initial and target orientations and the math used to determine these orientations, as well as axes that are aligned or misaligned with either extrinsic or intrinsic coordinate systems. In boxes (1) and (2) all initial orientations are the same. In box (1) the rotation axis is misaligned with either an extrinsic reference frame (top), or an intrinsic reference frame (bottom). In box (2) the rotation axis is aligned with these frames. In boxes (3) and (4) all target orientations are the same. In box (3) the rotation axis is misaligned with either an extrinsic reference frame (top), or an intrinsic reference frame (bottom). In box (4) the rotation axis is aligned with these frames. The notation of the initial and target orientations, and of the transition quaternions are in the inset.

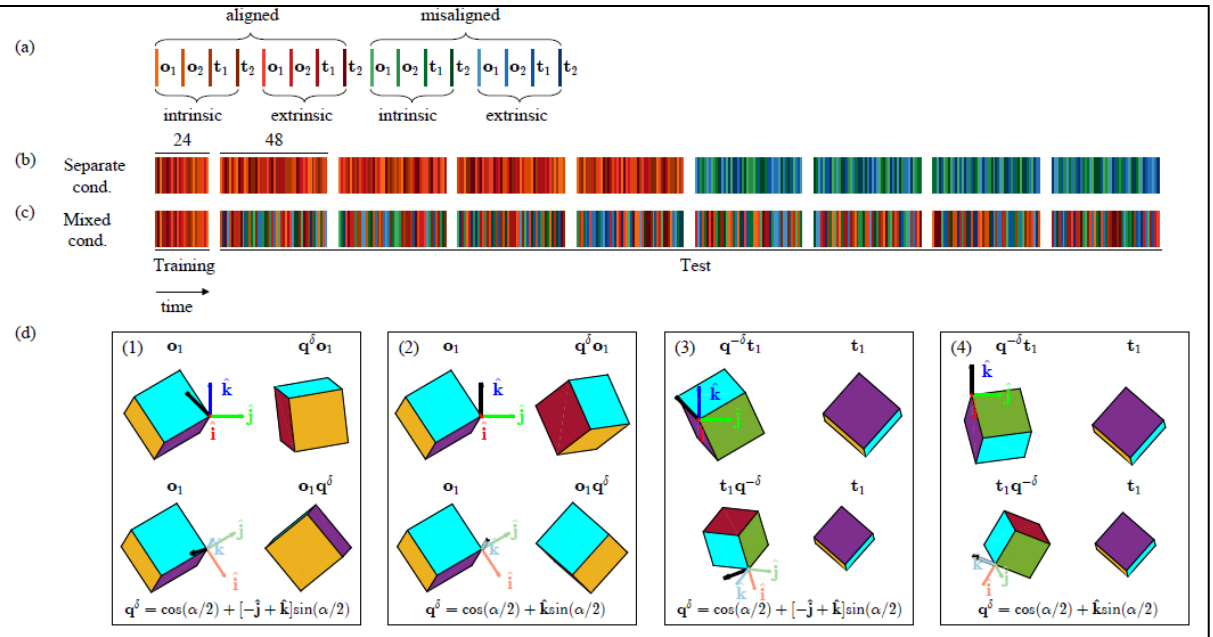

Fig 2. Experimental design and participants groups - experiment 1. The experiment consisted of two sessions: Training (24 trials) and Test (48×8 trials). In the Training session, all targets were presented by rotating the initial cursor orientation around an axis that was aligned to either an intrinsic or an extrinsic reference frame. The Test session of the Separate cond. group contained four trial sets with aligned axes followed by four trial sets with misaligned axes. The Test session of the Mixed cond. group contained eight trial sets with mixed aligned and misaligned axes. In both groups, half of the trials initiated from two fixed initial hand orientations ($\mathbf{o}_1$ and $\mathbf{o}_2 \in \mathbb{H}_1$), while the rest had two fixed target orientations ($\mathbf{t}_1$ and $\mathbf{t}_2 \in \mathbb{H}_1$). (a) Legend for the different experimental conditions of each trial. (b) The order of the trials in the Separate cond. group. (c) The order of the trials in the Mixed cond. group. (d) Examples of trials from the experiment. In each box, the left column shows the initial cursor orientation and the right column shows the respective target orientation (the notations of the initial cursor and target orientations, and the transition quaternion are in the inset; see Table 1 for more details). α is the rotation angle, and $\mathbf{q}^{-\delta}$ is the inverse of the transition quaternion. The top row shows trials constructed using rotation axes that are either misaligned (box 1 and 3) or aligned (box 2 and 4) with an extrinsic reference frame (dark red, green, and blue arrows). The bottom row shows trials constructed using rotation axes that are either misaligned (box 1 and 3) or aligned (box 2 and 4) with an intrinsic reference frame (light red, green, and blue arrows).

2. Expression using the quaternion is intelligent and impressive, but I am not sure how this expression is common in the motor learning research field. Thinking about the movement pattern from quaternions might be very hard. Displaying visual examples of the initial and target orientations for several conditions would help understand the difference between aligned and misaligned axes and between intrinsic and extrinsic rotations.

We added examples of the trial's conditions in Fig. 2d (see comment 1 for explanation). We agree that the quaternions are difficult to visualize which likely made them less commonly used in motor learning, but we believe that their benefits can advance the understanding of objects manipulation. We added a short discussion on the advantages and disadvantages of different representations of orientation, and explained the choice of quaternions:

"Our computational analysis design is based on the assumption that the nervous system represents the orientation of three-dimensional rigid bodies as quaternions. Quaternions are advantageous compared to other representations as their conversion to rotation matrices is nearly unambiguous. However, each rotation can be converted into two quaternions (q and -q). They also allow to define geodesics (see the Mathematical formulation section). However, quaternions may seem less intuitive compared to other

representation, and are hard to visualize, as they are four-dimensional objects. Other representations of orientation include the helical axis representation which describes a moving rigid body as translating and rotating around an axis. Yet, helical axes are more sensitive to measurement errors than quaternions [50]. Euler angles are a parameterization of the rotational space using a series of rotations about three mutually orthogonal axes. Euler angles representation suffers from several disadvantages, among them is the ambiguity in conversion from a rotation matrix to a set of Euler angles, which impose challenges on defining metrics [51], and therefore geodesics. In addition, Euler angles suffer from Gimbal lock - the loss of one rotational degree of freedom when two rotations act about the same axis. The current experiment does not allow for differentiating between different representations of orientation in the nervous system. We chose the quaternions because they provide a convenient computational framework that facilitates further studies, as we showed in the second experiment."

3. Did not the author observe the learning effect in Experiment 1? Performing the task in different condition seems not easy as represented by the larger QGS (Fig.6).

We did not observe a learning effect in experiment 1. We conducted a Wilcoxon rank sum test for the equality of the medians of the QGS of the first 10 trials and the last 10 trials of the Test session. We also computed the absolute difference in QGS and saw no large effects.

This analysis is described in the statistical analysis section:

"To check whether participants changed their QGS throughout the experiment, we conducted a Wilcoxon rank sum test for the equality of the medians of the geodicity scores of the first 10 trials and the last 10 trials of the Test session. To check for large effects, we computed the median of the absolute QGS differences over participants in each group."

We also added Fig. 7 in the results (shown below), which shows the time course of the QGS values in both groups (bold curves – median over participants, shaded curves – curve for each participant), and reported the results of the analysis:

"The QGS was not largely reduced throughout the experiment. Fig. 7 shows the time course of the QGS values in the Separate cond. group and in the Mixed cond. group. We tested whether participants changed the geodicity of their rotation in median throughout the Test session using a Wilcoxon rank sum test. Participants in both groups did not largely reduce their QGS (Mixed cond.: $p = 0.9$, mean diff. = 0.02, Separate cond.: $p = 0.07$, mean diff. = 0.29). This result was expected, as the orientation-matching task is a natural motor action that is performed on a regular basis."

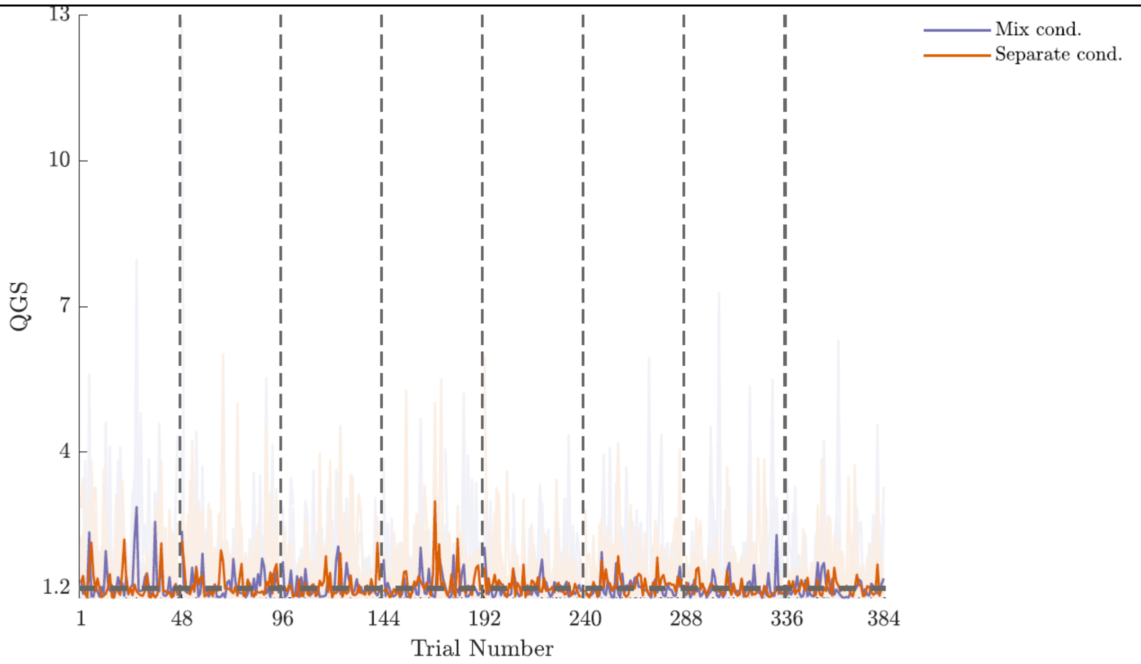

Fig 7. The time course of the QGS values. The QGS values were not largely reduced from the beginning of the experiment to its end. The light shaded curves show the time courses of the QGS values of each participant, and the bold curves show the time course of the median QGS in each group. The horizontal dashed gray line at QGS = 1.2 is the upper limit of the region of interest. The vertical dashed gray lines separate between the different sets of trials.

4. This might be related to the point #3 described above. In Exp.1, there were no statistical differences in the QGS values among various conditions. However, was it possible that the absence of significant difference resulted from the interference between intrinsic and extrinsic rotations? I was interested in how the large QGS trial took place. It seems that the large QGC would be generated by the transition from intrinsic to extrinsic rotation and vice versa?

As suggested, we looked on the transition between different conditions. Specifically we looked at transitions between trials with aligned-intrinsic axes and aligned-extrinsic axes, and between trials with misaligned-intrinsic axes and misaligned-extrinsic axes. We did not see that the QGS was larger in any of the specific transitions compared to the overall distributions of the QGS. We mention it in the results:

"One might expect the large QGS trials to be a result of the transition between rotating around axes that are aligned or misaligned with an extrinsic reference frame and axes that are aligned or misaligned with an intrinsic reference frame. A visual examination of the data showed that the large QGS trials are not more common when such interference occurs, which reinforces the robustness of the QGS to the conditions of the trials."

To make this point convincing, we provide here the distributions of the QGS in trials that took place after such transition occurred, and in trials that took place after such transition did not occure (blue – from extrinsic to intrinsic, orange – from intrinsic to extrinsic, and yellow – the rest of the trials). The analysis include four figures – two for each aligned axes (one figure for each group), and two for misaligned axes (one figure for each group).

We chose to leave this analysis out of the manuscript, but if the reviewer / editor advise otherwise we will be happy to add it.

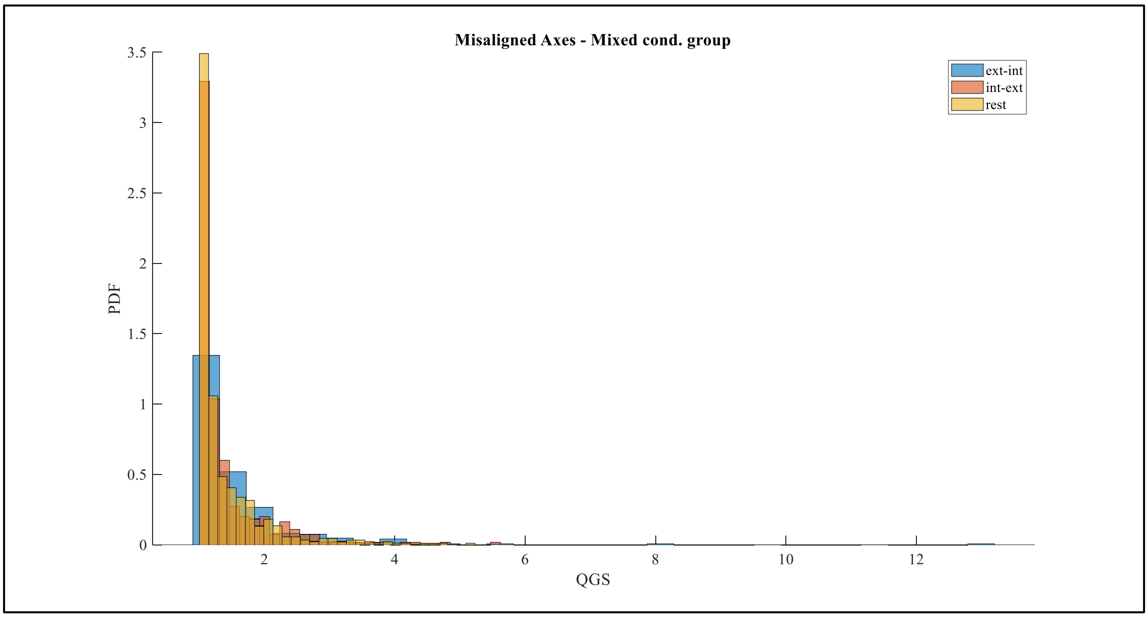

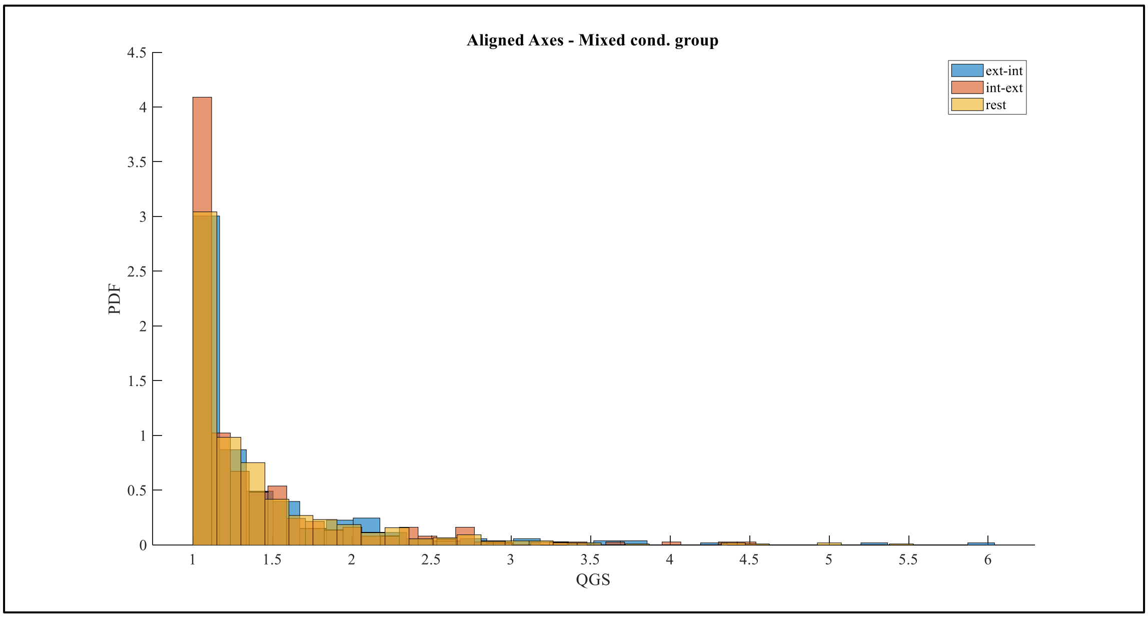

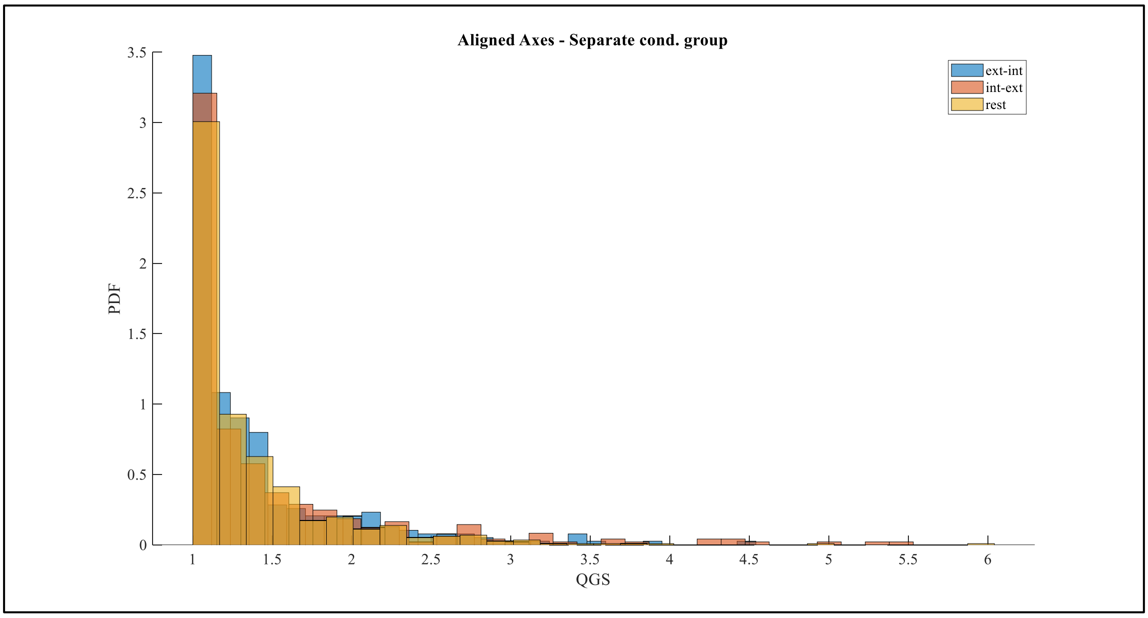

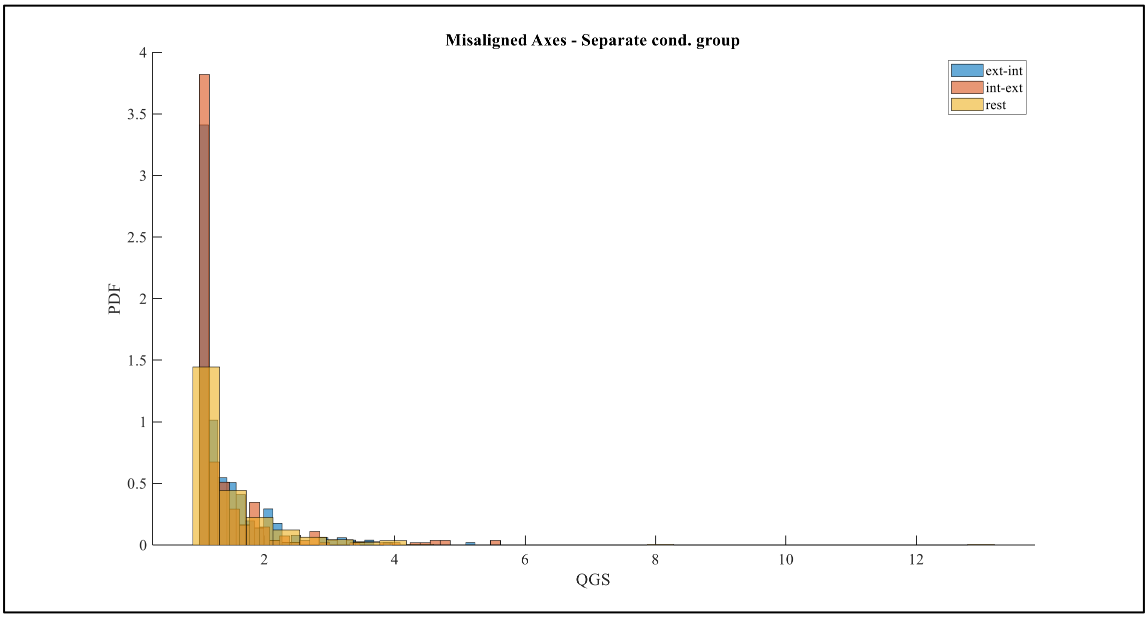

5. I wondered how stable the baseline trials in Exp.2 were. The QGS values were maintained low? If the movement in the baseline was not stable enough (as shown in Fig.6), the data of adaptation experiment was not reliable.

This is an important comment, as the adaptation would not be reliable if the baseline trials where not geodetic (i.e., with QGS close to 1). Therefore, we computed the QGS values of the baseline trials, as well as their median and its 95% bootstrap confidence interval. We also computed the 5% and 95% percentiles of the QGS and found that the trials were performed by closely following a geodesic, as the QGS was narrowly distributed near the ideal geodesic.

First, we mention this analysis in the data analysis section:

"To support the reliability of the adaptation analysis, we ensured that the participants did rotate their hand along a geodesic in the BL trials using the QGS values, computed from the time of trial initiation until the time of peak angular speed."

Then, we describe the statistical analysis:

"To ensure that the BL trials were geodetic enough, and that their geodicity was stable, we computed the median QGS value for each group, as well as the 95% bootstrap CI for the median QGS, and the 5% and 95% percentiles of the QGS."

Finally, we report the results of the analysis in the results section:

"Participants performed geodetic rotations in the BL sessions. The reliability of the adaptation analysis highly depends on the geodicity of the rotations in the BL sessions. In all groups, the median QGS of all trials and participants was near 1 and had narrow CI (Extrinsic: median = 1.0081 [1.0074,1.0092], Intrinsic: median = 1.0075 [1.0069,1.0082], Control: median = 1.0067 [1.0062,1.0074]). Moreover, the QGS was stable and narrowly distributed as indicated by the 5% and 95% percentiles (Extrinsic: [1.0011,1.1637], Intrinsic: [1.0013,1.1621], Control: [1.0011,1.0914]). Therefore, the subsequent analysis is reliable."

<u>Minor points</u>:

Line 383 "Early Transfer": Figure 4 used "First Transfer".

Mistake fixed.

Line 555 "Fig.8a": Fig.8a does not show any data of the last BL1 and that of the first TRN. Fig.8a should be Fig.8c?

Mistake fixed. The text now refers to Fig. 9, which shows the comparisons between the aiming axes.

Lines 560-562: The sentence seems inconsistent with the data. Fig.8c shows no significant difference in the aiming direction between the last BL1 and the first TRN.

Mistake fixed.

Line 573 "Fig.8a": Again, Fig.8 does not show any data of the first TRN and that of the last TRN. Fig.8a should be Fig.8c?

Mistake fixed.

Line 604 "Fig.8a": Fig.8a should be Fig.8c.

Mistake fixed.
